# Comparative Transcriptome Analysis of White and Purple Potato to Identify Genes Involved in Anthocyanin Biosynthesis

**DOI:** 10.1371/journal.pone.0129148

**Published:** 2015-06-08

**Authors:** Yuhui Liu, Kui Lin-Wang, Cecilia Deng, Ben Warran, Li Wang, Bin Yu, Hongyu Yang, Jing Wang, Richard V. Espley, Junlian Zhang, Di Wang, Andrew C. Allan

**Affiliations:** 1 Gansu Key Laboratory of Crop Improvement and Germplasm Enhancement, Gansu Agricultural University, Lanzhou, China; 2 The New Zealand Institute for Plant & Food Research Limited (Plant & Food Research) Mt Albert, Auckland, New Zealand; 3 College of Life Science and Technology, Gansu Agricultural University, Lanzhou, China; 4 College of Horticulture, Gansu Agricultural University, Lanzhou, China; 5 College of Food Science and Engineering, Gansu Agricultural University, Lanzhou, China; 6 School of Biological Sciences, University of Auckland, Auckland, New Zealand; NARO Institute of Fruit Tree Science, JAPAN

## Abstract

**Introduction:**

The potato (*Solanum tuberosum*) cultivar ‘Xin Daping’ is tetraploid with white skin and white flesh, while the cultivar ‘Hei Meiren’ is also tetraploid with purple skin and purple flesh. Comparative transcriptome analysis of white and purple cultivars was carried out using high-throughput RNA sequencing in order to further understand the mechanism of anthocyanin biosynthesis in potato.

**Methods and Results:**

By aligning transcript reads to the recently published diploid potato genome and *de novo* assembly, 209 million paired-end Illumina RNA-seq reads from these tetraploid cultivars were assembled on to 60,930 transcripts, of which 27,754 (45.55%) are novel transcripts and 9393 alternative transcripts. Using a comparison of the RNA-sequence datasets, multiple versions of the genes encoding anthocyanin biosynthetic steps and regulatory transcription factors were identified. Other novel genes potentially involved in anthocyanin biosynthesis in potato tubers were also discovered. Real-time qPCR validation of candidate genes revealed good correlation with the transcriptome data. SNPs (Single Nucleotide Polymorphism) and indels were predicted and validated for the transcription factors MYB *AN1* and *bHLH1* and the biosynthetic gene anthocyanidin 3-O-glucosyltransferase (*UFGT*).

**Conclusions:**

These results contribute to our understanding of the molecular mechanism of white and purple potato development, by identifying differential responses of biosynthetic gene family members together with the variation in structural genes and transcription factors in this highly heterozygous crop. This provides an excellent platform and resource for future genetic and functional genomic research.

## Introduction

Potato (*Solanum tuberosum* L.) is one of the world’s most important food crops, and is recognized as a source of health-promoting antioxidants for the human diet [1]. Potato tubers contain significant amounts of polyphenols. Anthocyanins are the predominant group of visible polyphenols that comprise the red, purple, and blue pigmentation of potato [2–4]. As well as playing important physiological roles in plant response to stress and acting as attractants for pollination and seed dispersal, anthocyanins have high free radical scavenging activity, anti-inflammatory and anti-microbial properties, and dietary consumption has been associated with a reduced incidence of cardiovascular diseases, cancers, neurodegenerative diseases, osteoporosis, or diabetes [5–7]. Pigmented potato genotypes have been shown to contain significantly higher levels of anthocyanins and antioxidant activity; especially cultivars with purple and red skin and/or flesh, compared to those with white and yellow tubers [4–7]. Therefore, a high anthocyanin potato has potential as a new cultivar with enhanced health benefits.

In many plant species, the anthocyanin biosynthetic pathway and its regulation has been elucidated. The genes that encode flavonoid biosynthetic steps and regulatory genes have also been cloned [8, 9]. The biosynthesis of anthocyanin pigments is regulated at the transcriptional level by a MYB-bHLH-WD40 (MBW) transcription factor (TF) complex [10, 11], composed of TFs from the R2R3-MYB, basic Helix-Loop-Helix (bHLH) and WD40 classes [12, 13]. MYB TFs can be classified into three subfamilies based on the number of highly conserved imperfect repeats in the DNA-binding domain including R3 MYB (MYB1R) with one repeat, R2R3 MYB with two repeats, and R1R2R3 MYB (MYB3R) with three repeats [14]. Those associated with up-regulation of the anthocyanin pathway are R2R3 MYBs. The first plant protein studied with a bHLH domain was *Lc* which is involved in the control of flavonoid/anthocyanin biosynthesis in maize [15]. The first two hundred amino acids of this bHLH protein are required to interact with the MYB transcription factor partner, while the C-terminal of the protein interacts with WD40 proteins [16]. The C-terminal ACT-like domain facilitates binding of the MYB to the promoter [17]. WD proteins have four to eight imperfect tandem repeats and interact with other proteins through the WD repeat region [18, 19].

Genes involved in the regulation of the anthocyanin pathway have been identified in many different species, for example *AN1* and *AN2* in *Petunia* [20, 21]; *C1* and *P1* in *Zea mays* [22, 23]; *Rosea1* and *Delila* in *Antirrhinum* [24, 25]; *PAP1* and *TTG8* in Arabidopsis [26]; *MYB1* and *MdMYB10* in apples [27, 28]; and *VvMYBA* in grape [29]. In apple, two candidate bHLH transcription cofactors (bHLH3 and bHLH33) are also needed for activating promoters of anthocyanin biosynthetic genes and MYB10 autoregulation [30].

In potato, previous studies have found that the biosynthesis of anthocyanin pigments in the periderm of the tuber is controlled in part by three loci, D, P, and R. Genetic evidence based on the co-localization in the potato genetic map of R, P and D indicated these loci encode dihydroflavonol 4-reductase (*DFR*), flavonoid 3’, 5’-hydroxylase (*F3’5’H*) and an R2R3 MYB transcription factor designated *AN1*[31].

The recent development of next-generation sequencing technologies can generate sequences on an unprecedented scale with a markedly reduced cost compared with traditional technologies [32]. Next-generation RNA-sequencing (RNA-Seq) has rapidly replaced microarrays as an approach to profile transcriptomes in a high-throughput way [33]. It allows detection of transcripts with low abundance, identifies novel transcript units, and reveals their differential expression between different samples [34, 35].

To date, there have been no reports of using RNA-Seq technology to analyze the control of pigmentation in different potato cultivars. The availability of the complete genome sequence of the doubled monoploid *Solanum tubersosum* clone DM1-3 516R44 [36] has made RNA-seq more informative. In this study, we used next-generation sequencing technology and bioinformatics tools to analyze the transcriptome of tetraploid white and purple potato cultivars, by both *de novo* assembly of transcripts and alignment to the published diploid potato genome to analyse differentially expressed genes related to anthocyanin biosynthesis between the cultivars and discovered new versions of the pathway genes and potential transcription factors. In addition, putative SNPs were identified and validated in *AN1 bHLH1* and *UFGT*. According to the generated RNA-seq datasets, there are significant differences in expression among genes involved in anthocyanin biosynthesis. These RNA-seq datasets enhance the available transcriptome data of potato, and improves our understanding of variations in tuber color to identify important genes for anthocyanin biosynthesis in potato.

## Materials and Methods

### Plant material, RNA extraction, library construction and RNA sequencing

**Fig 1:**
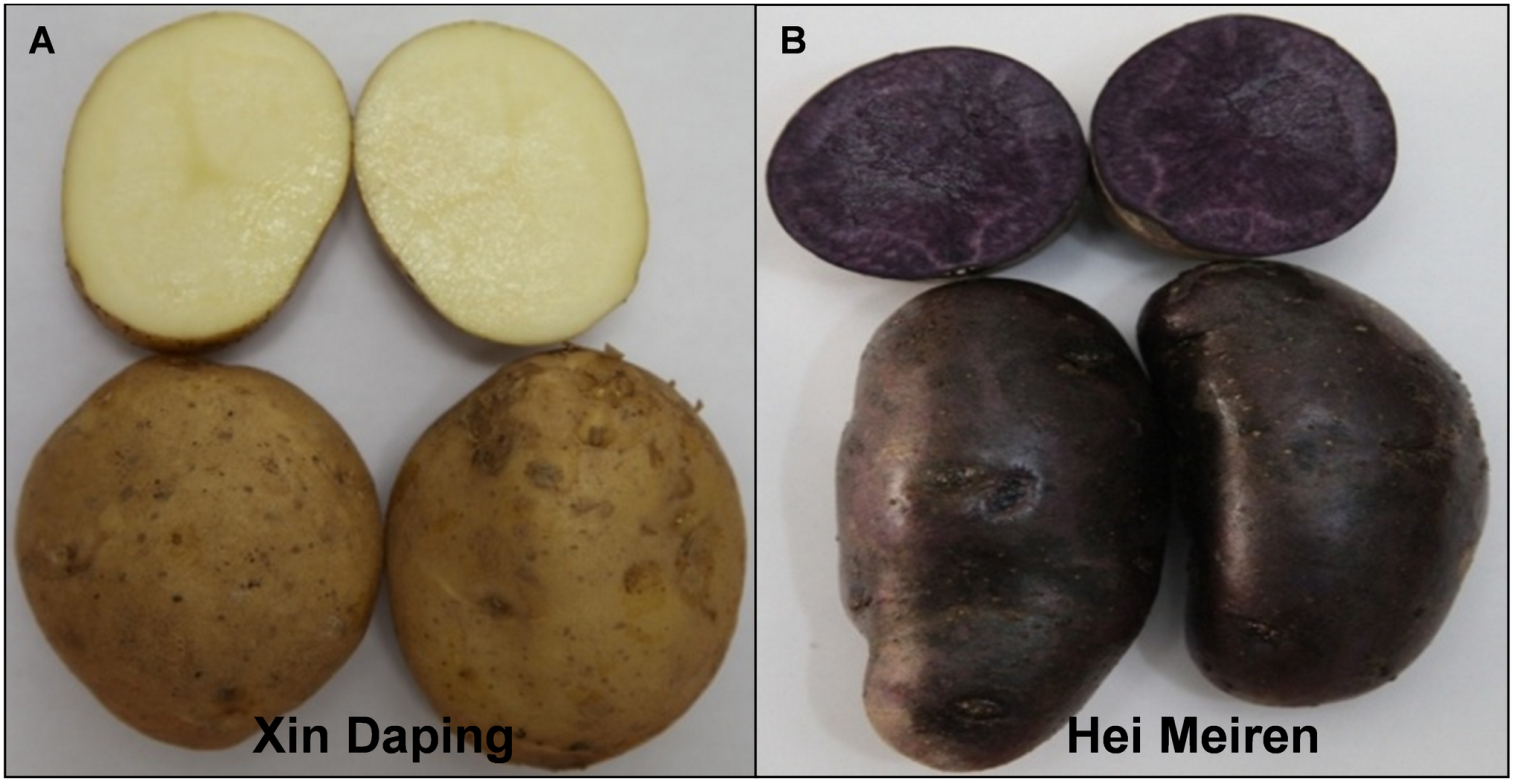
Tubers of *Solanum tuberosum* cultivars. (A) white skin and white flesh tetraploid cultivar ‘Xin Daping’, (B) purple skin and purple flesh tetraploid cultivar ‘Hei Meiren’.

A purple potato (*Solanum tuberosum* L.) cultivar ‘Hei Meiren’ (purple skin and purple flesh), a white potato cultivar ‘Xin Daping’ (white skin and white flesh) (Fig 1), two red potato cultivars ‘Gannongshu NO.5’ (red skin and white flesh) and ‘Qinshu NO.9’ (red skin, white flesh and red vascular ring) (S1 Fig) were grown in a greenhouse at Gansu Agricultural University, China. Three potato cultivars ‘Agria’ (white skin and white flesh), ‘Red Jackets’ (red skin and white flesh) and ‘Heather’ (purple skin and white flesh) (S1 Fig) were bought in a local supermarket, New Zealand. Five fresh tubers (diameter 4-5cm) were harvested, and cleaned with sterilized water. Skin tissue was carefully separated from cortex tissue using a scalpel to minimize flesh contamination. The skin and flesh of these potatoes were then immediately frozen in liquid nitrogen and stored in a -80°C freezer for later use.

Total RNA was extracted from purple skin and purple flesh of ‘Hei Meiren’ and white skin and white flesh of ‘Xin Daping’ using the PureLink Plant RNA Reagent Kit (Invitrogen, USA) according to the manufacturer's instructions. The RNA was quality checked and quantified using a Nanodrop ND-1000 (Nanodrop Technologies, USA). Enrichment of mRNA, fragment interruption, addition of adapters, size selection, PCR amplification and RNA-Seq were performed by Beijing Genome Institute (BGI; Shenzhen, China). The mRNA was separated from total RNA using oligo (dT) magnetic beads (Invitrogen, USA), then fragmented into short fragments using fragmentation buffer. cDNA was synthesized using the mRNA fragments as templates. Short fragments were purified and resolved with EB buffer for end repair and single nucleotide A (adenine) addition. Short fragments (200±20bp) were connected to adapters, suitable fragments are selected for the PCR amplification as templates according to agarose gel electrophoresis results, then an Agilent 2100 Bioanalyzer and ABI StepOnePlus Real-Time PCR Systems were used in quantification and qualification of the sample library. In total, there were 4 RNA-Seq libraries constructed—purple skin (PS), purple flesh (PF), white skin (WS) and white flesh (WF)—using the same protocol. Finally, an aliquot from each of the libraries was barcoded, pooled and sequenced using Illumina HiSeq 2000 in paired-end (PE) mode. The remaining RNA was used for real-time quantitative PCR (qPCR) verification. The RNA-seq dataset is available at the NCBI Sequence Read Archive (SRA) with the accession number: SRP036626.

### RNA Data filtering and *de novo* transcriptome assembly

Raw RNA sequencing reads passing BGI’s quality control (QC) were 100bp long in length. According to the FastQC (http://www.bioinformatics.babraham.ac.uk/projects/fastqc/) report, the reads were further trimmed to 75bp long, with 15bp removed from 5’ and 10bp from 3’. Trimmed reads with phred scores less than 20 and uncertain bases N were discarded from the analysis. *De novo* transcriptome assembly was performed for each cultivar (‘Hei Meiren’ with PS and PF, ‘Xin Daping’ with WS and WF) using Trinity version r20131110 (http://trinityrnaseq.sourceforge.net/). Transcriptome assembly completeness was assessed using cegma_v2.4.010312 (http://korflab.ucdavis.edu/datasets/cegma/). These two assemblies were merged with PGSC_DM_v3.4 gene models downloaded from the Potato Genome Sequencing Consortium (PGSC, http://solanaceae.plantbiology.msu.edu/pgsc_download.shtml, [36]) to create the consensus potato transcriptome reference using EvidentialGene VERSION 2013.07.27 (http://arthropods.eugenes.org/EvidentialGene/).

### Differentially gene expression test

Cleaned reads from each of the 4 RNA-Seq libraries were mapped to the consensus potato transcriptome reference sequences using bowtie2-2.2.1 (http://bowtie-bio.sourceforge.net/bowtie2/index.shtml) with a maximum fragment size of 500 and maximum of 1 mismatch in seed region of 20 bases. Raw read counts were extracted from the sorted alignment files. For differential expression test (DET), we compared three conditions: WF vs. PF; WS vs. WS; White (S & F) vs. Purple (S & F). For each comparison, genes with low read counts per million (cpm < 1) that didn’t represent sufficient statistic significance were excluded from the DET analysis. The libraries were digitally normalized after filtering and the DET was completed using EdgeR 3.24 (http://www.bioconductor.org/packages/release/bioc/html/edgeR.html) [37] and gplots 2.14 (http://cran.r-project.org/web/packages/gplots/index.html) in R 3.0.1 (http://www.r-project.org/) environment. Genes with FDR < 0.05 and the absolute value of logFC (log2 fold change) not less than 1.0 were considered as highly differentially expressed genes (DEGs). The gene expression unit was calculated using the RPKM [38] method (Reads per kilobase transcriptome per million mapped reads).

### Annotation and predicted CDS

Identified differentially expressed transcripts were annotated using MapMen Mercator Web Service (http://mapman.gabipd.org/web/guest/app/mercator) by searching the Arabidopsis TAIR proteins (TAIR10), SwissProt/UniProt Plant Proteins, Clusters of orthologous eucaryotic genes database (KOG) and Use conserved domain database (CDD), with a value of blast cut-off of 80. The final description for each novel transcript that was assembled from our RNA-Seq data but not predicted in the PGSC gene set was based on a series of searches as well as the ratio lengths to the HSP (highest-scoring segment pairs) from Blast. The complete Open Reading Frame (ORF) was predicted using the BioPerl (http://www.bioperl.org/).

### Gene ontology functional enrichment and pathway analysis of DEGs

The DEGs were mapped to GO terms in the GO database (http://www.geneontology.org/) to calculate gene numbers for every term. The hypergeometric test was then used to find significantly enriched GO terms in the input list of DEGs, based on 'GO::TermFinder' (http://smd.stanford.edu/help/GO-TermFinder/GO_TermFinder_help.shtml/). GO terms conforming to p-value through Bonferroni Correction **≤** 0.05 were defined as significantly enriched GO terms. KEGG [39] (http://www.genome.jp/kegg/) is used to perform pathway enrichment analysis of DEGs. MapMan software (version 3.6.0) was used to display expression profiles at the pathway level [40]. The expression profiles of the metabolic pathways can be viewed by a discrete signal visualized using different types (blue and red) and intensity of color.

### Real-time PCR (qPCR) quantitative analysis

For qPCR analysis, total RNA from skin and flesh of white cultivars ‘Xin Daping’ and ‘Agria’, purple cultivar ‘Hei Meiren’ and ‘Heather’, red cultivars ‘Gannongshu NO.5’, ‘Qinshu NO.9’ and ‘Red Jackets’ were extracted as described above. Each RNA sample was subjected to DNase digestion (Takara, Dalian, China) to remove any remaining DNA and first strand cDNA synthesis was carried out using oligo dT according to the manufacturer’s instructions (SuperScript III, Invitrogen, USA). cDNA was diluted twenty-fold and used as the template for qPCR. All the primer sequences for 4-coumarate-CoA ligase (*4CL*), flavanone 3 beta-hydroxylase (*F3H*), flavonoid 3'-monooxygenase (*F3’H*), chalcone synthase (*CHS*), dihydroflavonol 4-reductase (*DFR*), flavonoid 3',5'-hydroxylase (*F3’5’H*), leucoanthocyanidin dioxygenase/anthocyanidin synthase (*LDOX/ANS*), flavonol synthase (*FLS*), anthocyanidin 3-O-glucosyltransferase (*UFGT*), transcription factors *AN1* ^*816*^ and *bHLH1* are listed in S1 Table. qPCR DNA amplification and analysis was carried out using the LightCycler System (Roche LightCycler 480, Roche), with LightCycler software version 4. The LightCycler FastStart SYBR Green Master Mix (Roche) was used and 10 μl of total reaction volume applied to all the reactions following the manufacturer’s method. qPCR conditions were 5 min at 95°C, followed by 40 cycles of 5 s at 95°C, 5 s at 60°C, and 10 s at 72°C, followed by 65°C to 95°C melting curve detection. The qPCR efficiency of each gene was obtained by analyzing the standard curve of a cDNA serial dilution of that gene. Relative abundance was calculated with the ΔC_T_ method using actin (accession number: X55752) for template normalization. Potato actin is widely used as a reference gene in qPCR analysis [19, 41, 42]. Error bars shown in qPCR data are technical replicates, representing the means ±SE of four replicate qPCR reactions. Statistical significance was determined by one-way ANOVA.

### SNPs and indel discovery

We used the nucleotide sequences of the published *UFGT1* (KP096267) from white skin of ‘Xin Daping’ cultivar, *AN1* (AY841127) from red skin of ‘Y83-1’ cultivar and *bHLH1* (JX848660) from purple tuber of ‘Magic Molly’ as the reference sequences for SNPs and indels discovery. Cleaned reads from the white and purple genotypes were mapped back to the references using Bowtie 2 with mapping criteria ‘very sensitive’. Alignments with mapping quality less than 1 were removed from the BAM files using SAMTOOLS (http://samtools.sourceforge.net/) and custom BASH script. The reference and the filtered alignment BAM files were loaded to the IGV 2.3.25 (http://www.broadinstitute.org/igv/) for visualization. Putative SNPs and indels were detected with criteria of the minimum frequency greater than 10%.

### SNPs validation and expression analysis of *UFGT* in tetraploid cultivars

Genomic DNA from the seven cultivars: white cultivars ‘Xin Daping’ and ‘Agria’, purple cultivar ‘Hei Meiren’ and ‘Heather’, red cultivars ‘Gannongshu NO.5’, ‘Qinshu NO.9’ and ‘Red Jackets’ was extracted as described above, and total RNA from skin and flesh of each cultivar was also extracted. First strand cDNA synthesis was carried out using oligo dT according to the manufacturer’s instructions. PCR amplifications were performed in 25 μl reaction volume applied to all the reactions following the manufacturer’s method. PCR conditions were 5 min at 95°C, followed by 35 cycles of 30 s at 95°C, 30 s at 57°C, and 84 s at 72°C, and a final extension at 72°C for 10 min. The PCR products were quantified using a Nanodrop ND-1000 spectrophotometer and quality was assayed on a 1% agarose gel. 200 ng of PCR products were digested with the restriction endonuclease *Eco*RI to confirm the SNPs discovered in RNA-seq data of ‘Xin Daping’ and ‘Hei Meiren’, then the allelic composition and expression of *UFGT* were detected in the seven cultivars.

## Results

### Total RNA sequencing and *de novo* transcriptome assembly

There were 53,426,530 (WS), 51,032,576 (WF), 51,184,258 (PS) and 53,618,074 (PF) high-quality sequencing reads left after trimming and filtering. *De novo* transcriptome assembly using Trinity built 149,821 (WS), 112,714 (WF), 127,719 (PS) and 109,065 (PF) transcript contigs for each sample type. The contig lengths ranged from 201 to 13,644 bp and the GC contents ranged from 44.54% to 45.97% (Table 1).

**Table 1:** Statistical summary of the *de novo* assembly for WS, WF, PS and PF four libraries.

	WS**	WF	PS	PF
Total Reads (million)	53	51	51	54
Total Mapped Reads	48* (91%)^#^	47 (92%	47 (92%)	51 (94%)
Total Unmapped Reads	4.9 (9%)	4.1 (8%)	3.9 (8%)	3.4 (6%)
Contig number	149,821	112,714	127,719	109,065
Minimum Contig length (bp)	201	201	201	201
Maximum Contig length (bp)	9,934	13,644	10,036	10,008
GC% content	44.55%	44.54%	45.4%	45.97%

*Represents number of reads (million),

^#^Represents percentage of reads (mapped or unmapped) to total reads.

**WS: white skin library, PS: purple skin library, WF: white flesh library, PF: purple flesh library.

Gene prediction for double monoploid (DM) potato was downloaded from PGSC (http://potato.plantbiology.msu.edu/index.shtml) and contained 56,218 gene models. Genes for tetraploid potato may be missed out in the DM potato gene prediction. *De novo* transcript assembly usually contains redundant, short, and in-complete transcript contigs. To overcome these problems, we merged the *de novo* transcripts (WS, WF, PS and PF) and PGSC genes using evigene-18-09-2013 (http://eugenes.org/EvidentialGene/evigene/). The resulting consensus reference (WP&PGSC) consisted of 60,930 primary transcripts (with the highest scores for alignments, protein quality and identity) and 9,393 alternative forms (Table 2). Approximately 27,000 (45.55%) in the primary set were novel transcripts that were not predicted in, and did not cluster with, the DM potato genes.

**Table 2:** Merged transcriptome assembly.

Data	Description	Transcripts in primary set*	Alternative set**
WS&WF	Merged transcripts from WS and WF	35,441	17,802
PS&PF	Merged transcripts from PS and PF	38,617	20,571
WP	Merged transcripts from WS&WF and PS&PF	47,196	44,780
WP&PGSC^#^	Merged primary transcripts assembled for WP and DM potato reference genome	60,930	9393

*Primary set; the best transcripts from the two input transcriptome sets with highest scores based on alignments, protein quality and identity.

**Alternative set contains alternative splice forms to the transcripts in the primary set.

^#^PGSC is ab inito gene prediction for doubled monoploid *S*. *tuberosum* clone DM1-3 (DM) and downloaded from http://potato.plantbiology.msu.edu/index.shtml.

**Fig 2:**
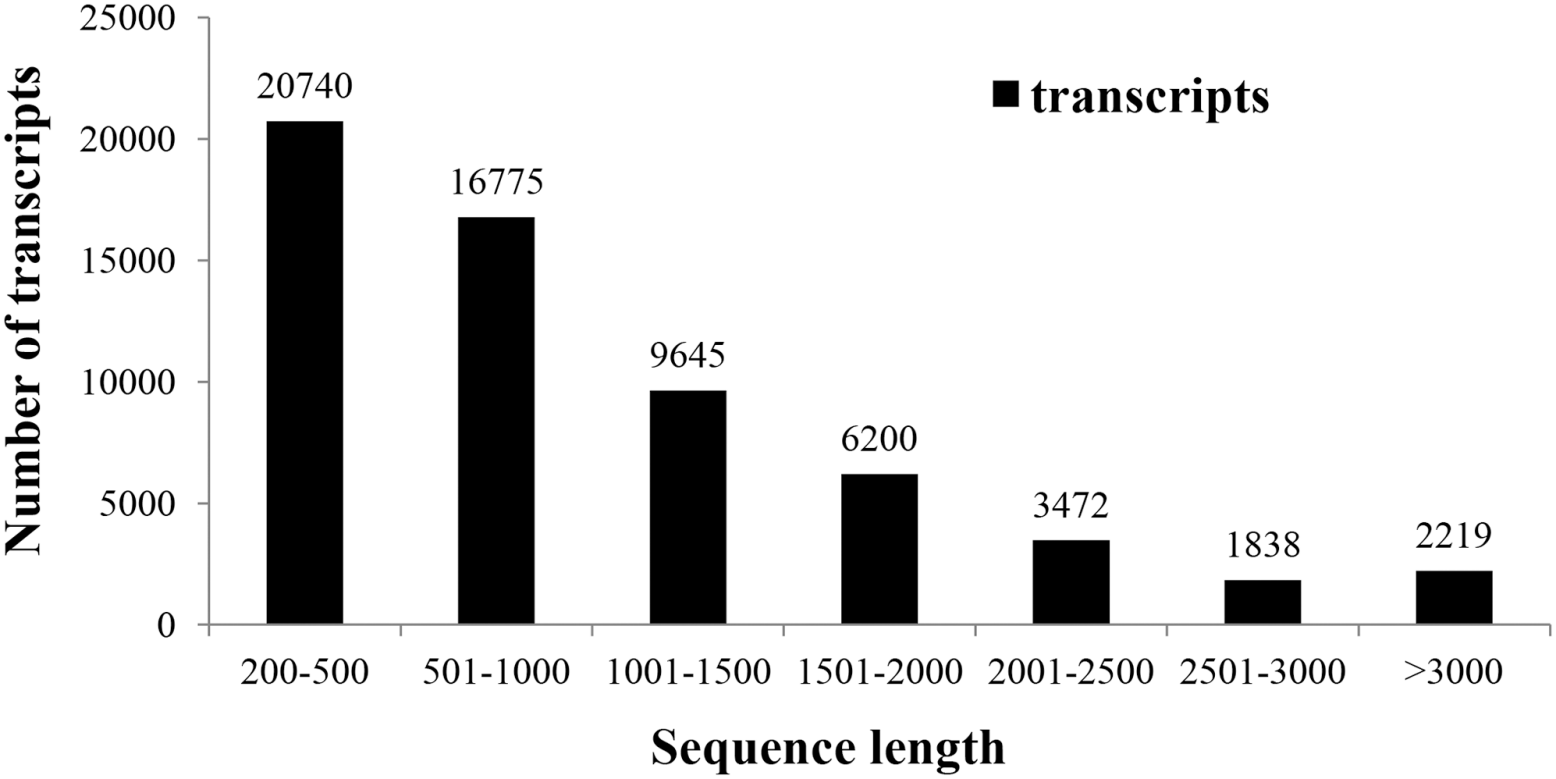
Length distribution of the 60,930 transcripts. Histogram of the sequence-length distribution of all transcripts including transcripts mapped to double monoploid (DM) potato reference genes (PGSC) and new transcripts assembled by *de novo* assembly. The x-axis indicates sequence sizes from 200 bp to >3000bp. The y-axis indicates the number of transcripts in the corresponding region of sequence length.

The majority of the transcripts in the consensus set were shorter than 2000 bp in size, with 20,740 (34.11%), 16,775 (27.53%), and 15,845 (26.01%) falling in the range 200bp to 500bp, 501 to 1000bp, and 1001 to 2000bp, respectively. Only 7529 (12.36%) transcripts were longer than 2000 bp (Fig 2).

High quality reads were mapped to the reference sequence (WP&PGSC) using Bowtie2, the total mapped reads were between 91–94%, while unmapped reads were between 6%–9% in the four libraries (Table 1). Complete ORFs were predicted from the 27,754 new transcripts, 19,652 (70.8%) were classified as possessing ORFs, the average ORF length is 644bp.

### Analysis, Functional Annotation and KEGG Classification of Differentially Expressed Genes

**Fig 3:**
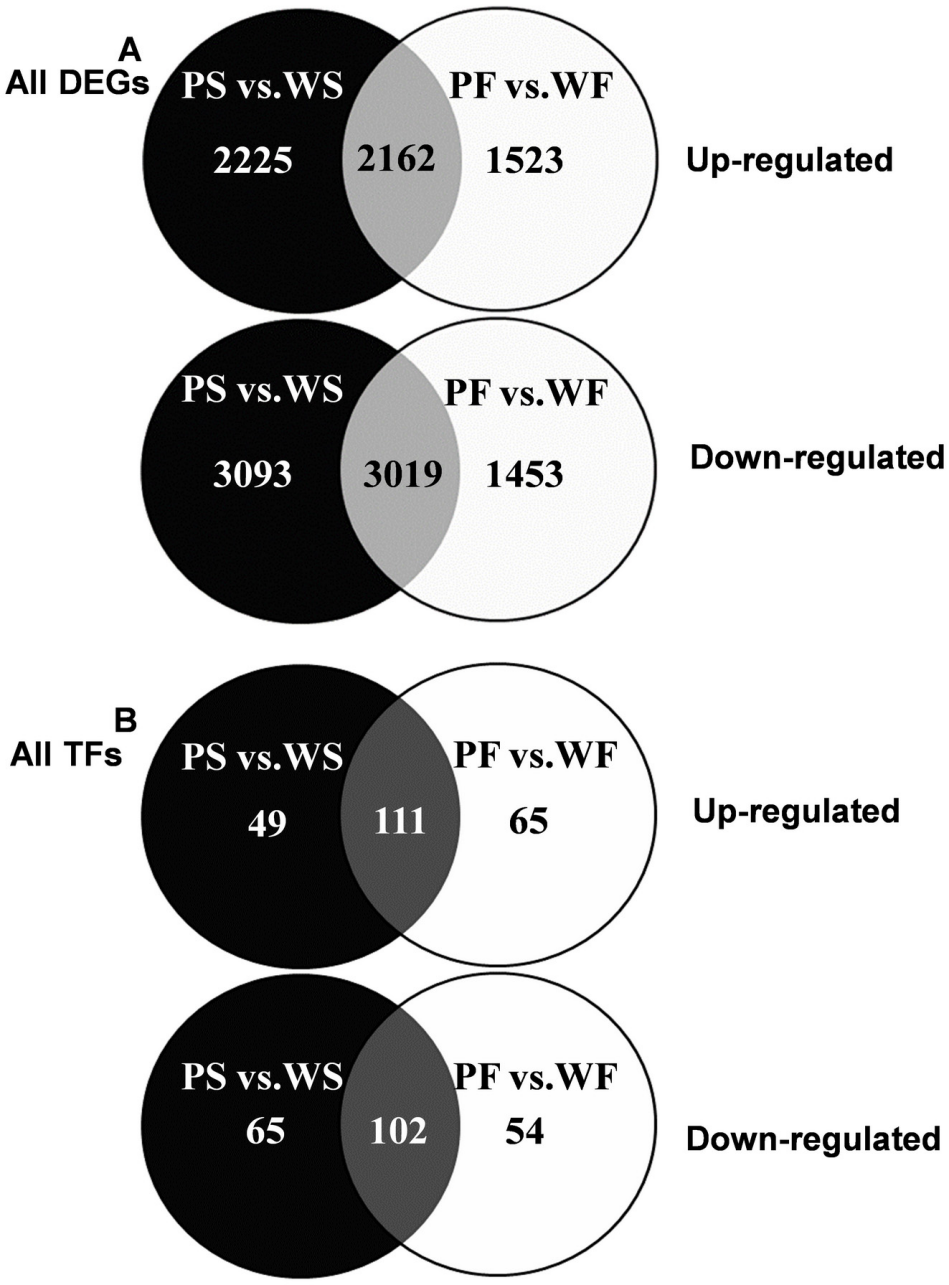
Venn diagram of all DEGs and all TFs. (A) The numbers of all DEGs both up-regulated and down-regulated in PS vs. WS library and PF vs. WF library. The black shading represents all up-regulated or down-regulated DEGs exclusively in PS vs.WS library, the white shading represents all up-regulated or down-regulated DEGs exclusively in PF vs.WF library, the light grey shading of overlapping regions represents consistent DEGs in both libraries. (B) The number of differentially expressed transcription factors (TFs). Shading as described for panel (A). (FDR < 0.05, absolute value of the logFC ≥ 1 and RPKM > 1 applied).

Analysis of DEGs between the four potato samples was used to ascertain genes potentially involved in anthocyanin accumulation. Using a FDR < 0.05, absolute value of the logFC ≥ 1 and RPKM > 1 as threshold values, 10,499 genes (7591 novel transcripts) between WS and PS libraries and 8,157 genes (5727 novel transcripts) between WF and PF libraries were found to be differentially expressed; 4,387 genes were up-regulated and 6,112 genes down-regulated in the PS vs. WS library, and 3,685 genes were up-regulated and 4,472 genes down-regulated in the PF vs. WF library (S2 Table and S2 Fig). 2,162 genes were up-regulated in both the PS vs. WS and PF vs. WF libraries. In addition, 3,019 genes were down-regulated in both PS vs. WS and PF vs. WF libraries (Fig 3). Less than 1% of the genes were expressed at more than 10,000 RPKM; 2.2%- 3.5% were expressed between 101–1,000 RPKM, while 97% of the genes were expressed at less than 100 RPKM (Table 3).

**Table 3:** The distribution of genes according to the expression level RPKM in four libraries.

RPKM	Gene number of WS	Gene number of WF	Gene number of PS	Gene number of PF
0–100	30890 (97.3%)	26617 (96.4%)	30749 (97.5%)	26829 (97.7%)
101–1,000	824 (2.7%)	955 (3.5%)	759(2.4%)	612 (2.2%)
1,001–10,000	25 (0.08%)	40 (0.1%)	22 (0.07%)	19 (0.07%)
>10,000	3 (0.009%)	2 (0.007%)	2 (0.006%)	1 (0.004%)
Total	31742	27614	31532	27461

We separated the DEGs into three groups according to fold change: 1–5 fold (1 ≤ logFC< 2.32, FDR ≤ 0.05), 5–10 fold (2.32 ≤ logFC < 3.32, FDR ≤ 0.05) and 10 ≥ fold (logFC ≥ 3.32, FDR ≤ 0.05) (Table 4). This shows that there are still over 1500 genes differentially expressed more than 10 fold between colored and non-colored tissue, in both flesh and skin of potato.

**Table 4:** All DEGs divided according to the folds of RPKM value between PS vs.WS and PF vs. WF libraries (FDR < 0.05, absolute value of the logFC ≥ 1 and RPKM > 1 applied).

Fold	PS vs. WS			PF vs. WF		
	Number	Upregulated	Downregulated	Number	Upregulated	Downregulated
1≤fold<5	6179	2705	3474	5359	2582	2777
5≤fold<10	1713	643	1070	1282	529	753
Fold≥10	2607	1039	1568	1516	574	942
Total numbers	10499	4387	6112	8157	3685	4472

The 13,318 novel differentially expressed transcripts in the between PS vs. WS library and PF vs. WF library were searched against the Arabidopsis TAIR proteins, SwissProt/UniProt Plant Proteins, KOG and CDD databases using BLASTx employing a cut-off e-value of 80 as the criterion for defining a significant hit. 4546 (59.9%) out of 7591 novel transcripts in PS vs. WS libraries and 3697 (64.6%) out of 5727 novel transcripts in PF vs. WF libraries showed significant BLASTx matches in the Arabidopsis TAIR proteins (TAIR10). In UniProtKB/Swiss-Prot database, 3209 (42.3%) out of 7591 and 2525 (44.1%) out of 5727 novel transcripts had BLAST hits to known proteins in PS vs. WS libraries and PF vs. WF libraries, respectively. All of the transcripts that had BLAST hits in UniProtKB/Swiss-Prot database also had the BLAST matches in the Arabidopsis TAIR proteins (TAIR10). There were 3045 (40.1%) novel transcripts in PS vs. WS libraries and 2030 (35.4%) novel transcripts in PF vs. WF libraries were not annotated.

KEGG is a pathway-related database, providing classification for biologically complex patterns. Among those differentially expressed genes with a KEGG pathway annotation, 6861 were identified in PS vs. WS library and mapped onto 126 KEGG pathways, while 5624 DEGs were identified in PF vs. WF library and mapped onto 125 KEGG pathways. These pathways included biosynthesis of secondary metabolites (ko01110), RNA transport (ko03013), flavonoid biosynthesis (ko00941), phenylpropanoid biosynthesis (ko00940), and plant hormone signal transduction (ko04075) (S3 Table). The differentially expressed genes were visualised within a metabolic map using the Mapman tool of known metabolic pathways for potato (S3 Fig). Pathways showing significant changes in gene expression between PS vs. WS and PF vs.WF libraries included cell signaling, cell wall metabolism, lipid metabolism, major and minor CHO metabolism and secondary metabolism. Interestingly, in the light-related transduction pathways, most of the genes were downregulated in the purple skin and purple flesh.

### Detection of genes related to anthocyanin biosynthesis pathway

**Fig 4:**
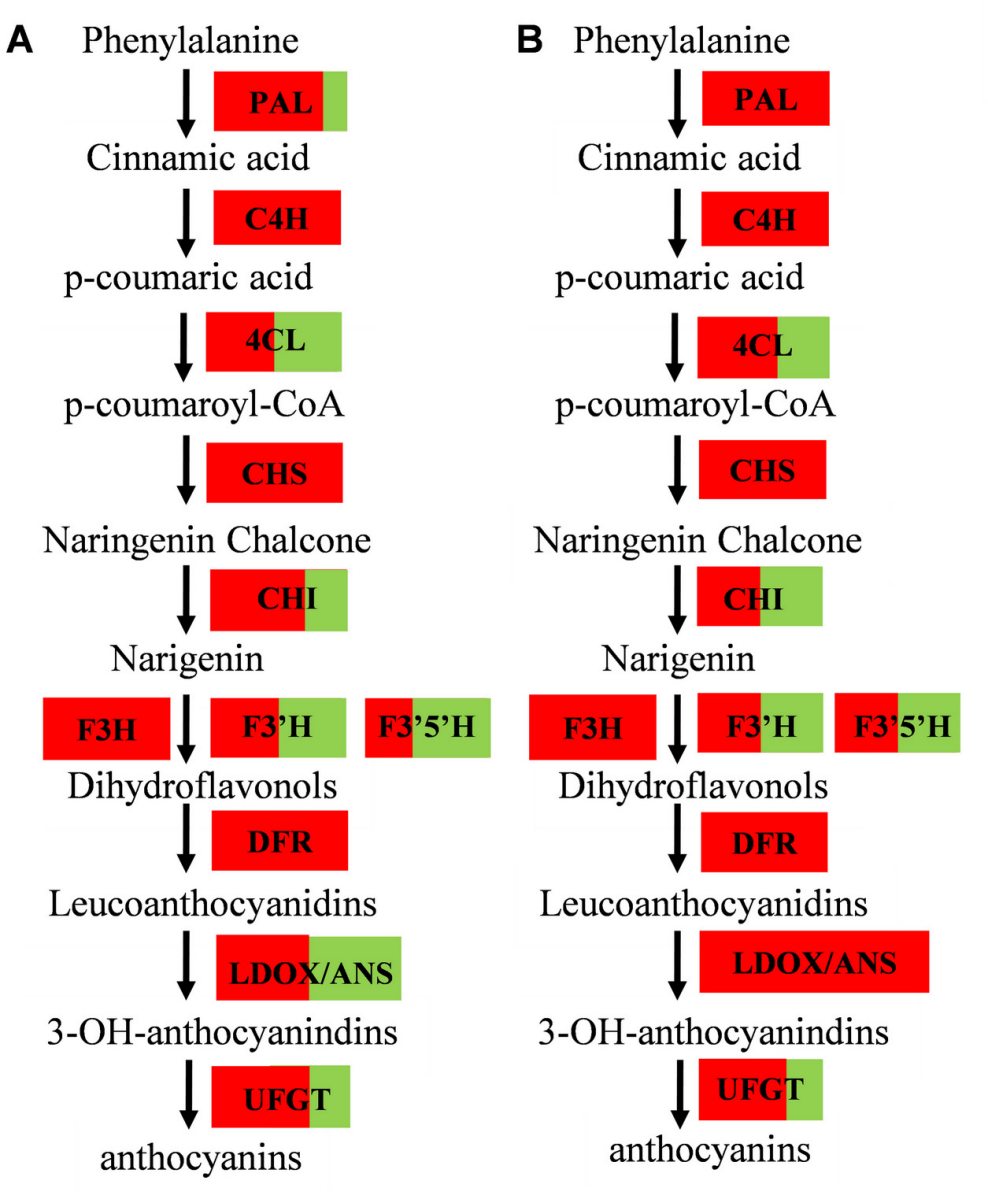
Anthocyanin biosynthesis compared between pigmented tissue and non-pigmented tissue in PS vs. WS libraries and PF vs. WF libraries. A red box indicates that multiple versions of the gene are all up-regulated. The box with both red and green indicates that some versions of the gene are up-regulated and the rest are down-regulated, and the ratio of red area to green area within the box indicates the ratio of the number of up-regulated versions to the number of down-regulated versions of that gene. (A) PS vs. WS library (B) PF vs. WF library.

Differentially expressed genes potentially involved in anthocyanin biosynthesis were identified. These included 48 genes in PS vs. WS library and 45 genes in PF vs. WF library including gene family members annotated as phenylalanine ammonia-lyase (*PAL*) (EC: 4.3.1.24), trans-cinnamate 4-monooxygenase (*C4H*) (EC:1.14.13.11), 4-coumarate-CoA ligase (*4CL*) (EC:6.2.1.12), chalcone synthase (*CHS*) (EC:2.3.1.74), chalcone isomerase (*CHI*) (EC:5.5.1.6), flavonoid 3'-monooxygenase (*F3’H*) (EC:1.14.13.21), flavanone 3 beta-hydroxylase (*F3H*) (EC:1.14.11.9), flavonoid 3',5'-hydroxylase (*F3’5’H*) (EC:1.14.13.88), dihydroflavonol 4-reductase (*DFR*) (EC:1.1.1.219), leucoanthocyanidin dioxygenase/anthocyanidin synthase (*LDOX/ANS*) (EC:1.14.11.19), anthocyanidin 3-O-glucosyltransferase (*UFGT*) (EC:2.4.1.115) and glutathione S-transferase (*GST*) (EC:2.5.1.18) (Table 5 and Fig 4).

**Table 5:** Homologous genes involved in anthocyanin biosynthesis in Arabidopsis and potatoes.

Pathway genes										
Arabidopsis gene		Potato gene	Gene ID^#^	Chr	Start	End	PS/WS^$^		PF/WF^$^	
							RPKM	logFC	RPKM	logFC
AtPAL1(AT2G37040)[65]		StPAL1-1	PGSC0003DMT400055531	9	5530471	5533348	-	-	2/1	1.56
		StPAL1-2	PGSC0003DMT400055488	9	5510117	5512923	39/19	1.33	3/1	1.76
		StPAL1-3	PGSC0003DMT400055489	9	5502194	5505885	325/42	3.23	16/4	4.31
		StPAL1-4	OkPFcomp53176_c0_seq11*				197/32	2.88	28/6	2.74
		StPAL1-5	OkPFcomp53176_c0_seq12*				116/19	2.86	16/4	2.59
		StPAL1-6	OkWScomp62786_c0_seq8				-	-	3/1	1.89
AtPAL2(AT3G53260)[66]		StPAL2-1	PGSC0003DMT400060308	5	51694756	51698709	-	-	2/0.3	3.65
		StPAL2-2	OkWScomp47869_c0_seq1*				1/0	-8.91	-	-
AtC4H(AT2G30490)[65]		StC4H	PGSC0003DMT400035590	5	43217169	43219535	3/0.6	2.42	1/0.5	1.95
At4CL1 (AT1G51680)[65]		St4CL1-1	PGSC0003DMT400037485	12	49327916	49340023	-	-	2/1	1.47
		St4CL1-2	OkPScomp55734_c1_seq3				-	-	4/2	1.63
At4CL2(AT3G21240)[65]		St4CL2	OkPScomp55734_c1_seq1				-	-	4/2	1.38
At4CL3(AT1G65060)[67]		St4CL3-1	PGSC0003DMT400008182	3	47072062	47077037	18/10	1.15	4/2	1.81
		St4CL3-2	OkPScomp55734_c1_seq5				8/5	1.12		-
		St4CL3-3	OkPScomp55734_c1_seq2				-	-	2/1	1.43
At4CL-5 like(AT1G20510)[68]		St4CL-5 like	PGSC0003DMT400075394	12	57354831	57359405	7/18	-1.03	5/22	-2.12
At4CL-9 like(AT5G63380)[68]		St4CL-9 like	PGSC0003DMT400039281	3	50885904	50891146	7/37	-2.09	3/14	-1.92
AtCHS(AT5G13930)[69]		StCHS1	PGSC0003DMT400049165	5	48886960	48888729	159/16	3.54	13/0.03	9.40
		StCHS2	PGSC0003DMT400076178	9	58356999	58359082	39/13	1.82	4/0.01	8.50
		StCHS3	OkPFcomp50370_c0_seq1				334/35	3.50	27/0.2	7.65
		StCHS4	OkWScomp62716_c1_seq1*				115/35	2.04	12/0.3	6.00
AtCHI(AT3G55120)[70]		StCHI1	PGSC0003DMT400030430	5	47476992	47479689	65/36	1.11	7/0.5	4.46
		StCHI2	OkPFcomp47529_c0_seq1				84/43	1.23	-	-
		StCHI3	OkWScomp63818_c2_seq1				2/6	-1.51	-	-
		StCHI4	OkWFcomp47607_c0_seq1				0.7/5	-2.61	0.3/4	-3.44
AtF3'H(AT5G07990)[71]		StF3'H1	PGSC0003DMT400063351	3	55232191	55236265	28/1	5.16	0.1/0.04	1.67
		StF3'H2	PGSC0003DMT400014232	4	60879015	60880900	2/0.1	4.13	2/0.07	5.36
		StF3'H3	OkPScomp54523_c2_seq4				78/3	4.96	58/1	6.03
		StF3'H4	PGSC0003DMT400033701	4	53669171	53672463	2/8	-1.94	0.2/1	-2.10
		StF3'H5	PGSC0003DMT400016263	4	7545873	7548307	15/146	-3.02	2/25	-3.12
		StF3'H6	PGSC0003DMT400004816	**	12427765	12431196	0.1/10	-6.98	-	-
AtF3H(AT3G51240)[72]		StF3H	PGSC0003DMT400009175	2	39368653	39370527	473/80	2.82	32/0.4	6.92
AtF3'5'H1 (AT4G12300)[73]		StF3'5'H1-1	OkPFcomp51142_c0_seq3				3/2	1.16	2/1	1.35
		StF3'5'H1-2	OkWScomp63366_c2_seq1				13/32	-1.06	-	-
		StF3'5'H1-3	PGSC0003DMT400012194	8	52875661	52883978	-	-	0.7/4	-1.66
AtF3'5'H2 (AT4G12320)[74]		StF3'5'H2-1	PGSC0003DMT400001124	11	39417579	39420629	238/71	2.01	16/0.2	7.12
		StF3'5'H2-2	PGSC0003DMT400012195	8	52871965	52881529	1/6	-1.94	0.7/2	-1.15
		StF3'5'H2-3	PGSC0003DMT400044122	**	36187416	36190626	0.3/1	-1.85	-	-
AtDFR(AT5G42800)[70]		StDFR1	PGSC0003DMT400009287	2	40293862	40297510	70/17	2.26	5/0.1	6.05
		StDFR2	PGSC0003DMT400087830	10	39522858	39523989	2/0.06	4.82	-	-
		StDFR3	OkPFcomp18287_c0_seq1				13/2	2.94	8/0.8	4.00
AtANS/AtLDOX (AT4G22880)[75]		AtANS/AtLDOX1	PGSC0003DMT400058554	8	53728302	53732590	173/35	2.57	12/0.4	5.63
		AtANS/AtLDOX2	OkWScomp60017_c0_seq2*				0.2/4	-3.87	-	-
AtUFGT1 (AT5G17050)[76]		StUFGT1	OkPFcomp47734_c0_seq1				45/24	1.16	5/0.2	5.15
AtUFGT2 (AT5G54060)[77]		StUFGT2	OkPFcomp53325_c0_seq5				16/2	3.16	7/0.7	4.02
		StUFGT3	OkPFcomp52560_c1_seq1				3/0.5	2.89	2/0.3	3.23
		StUFGT4	OkPFcomp53325_c0_seq2				18/4	2.34	8/1	3.25
		StUFGT5	OkPFcomp53325_c0_seq4				12/4	1.92	5/1	2.84
		StUFGT6	OkPFcomp49428_c0_seq1				180/60	1.85	185/70	1.96
		StUFGT7	OkWFcomp37394_c0_seq1*				17/7	1.56	14/6	1.91
		StUFGT8	PGSC0003DMT400041594	11	2150447	2152012	0.1/20	-7.37	0.1/2	-3.94
		StUFGT9	PGSC0003DMT400044262	9	55848257	55849693	1/18	-3.51	2/9	-1.74
		StUFGT10	OkWFcomp48966_c0_seq2*				3/49	-3.74	1/6	-2.10
		StUFGT11	OkWScomp63582_c0_seq7*				1/9	-2.52	0.7/6	-2.67
		StUFGT12	PGSC0003DMT400047460	3	46418857	46419888	0.4/9	-4.48	-	-
AtTT19(AT5G17220)[78]		StGST	PGSC0003DMT400043105	9	54418735	54424093	234/27	3.37	14/0.04	8.85
**Transcription factors**										
Published Potato gene	Chr	Potato gene	Gene ID^#^	Chr	Start	End	PS/WS^$^		PF/WF^$^	
							RPKM	logFC	RPKM	logFC
StAN1_816 StAN1-777 (AY841129/AY841127[45]	10	StAN1-1	OkPFcomp34237_c0_seq1				8/0.8	3.69	4/0.6	3.47
		StAN1-2	OkPScomp56435_c3_seq12*				13/64	-1.99	-	-
StMYBA1(JQ219855)	10	StMYBA1-1	OkWFcomp44929_c0_seq6				1/254	-7.30	0.05/19	-7.84
		StMYBA1-2	OkWScomp62330_c0_seq5*				0.5/51	-6.44	0/2	-8.37
		StMYBA1-3	OkPScomp56435_c3_seq8				53/340	-2.42	0.4/4	-2.79
StbHLH1(JX848660)[19]	9	StbHLH1	PGSC0003DMT400033569	9	47422301	47429221	22/4	2.84	1.7/0.3	3.12
StAN11(HQ599506)[79]	4	StAN11	PGSC0003DMT400094468	3	46418857	46419888	-	-	-	-

^#^: Gene ID beginning with “PGSC…” is the gene ID from Database of Potato Genome Sequencing Consortium (http://potatogenomics.plantbiology.msu.edu); while “OkPS…” is the gene ID form *de novo* assembly of purple skin library; purple flesh “OkPF…”; “OkWS white skin; and “OkWF…” white flesh.

*: partial sequences.

**: Unanchored Sequences.

^$^:—is for the genes that are differentially expressed (FDR < 0.05, absolute value of the logFC ≥ 1 and RPKM > 1 applied) in two libraries (PS/WS or PF/WF).

As shown in Table 5, four genes annotated as *CHS* were found to be differentially expressed in skin and flesh of the purple cultivar compared with those of the white cultivar, including *CHS1* (PGSC0003DMT400049165) located on chromosome 5 and a new transcript *CHS3* (OkPFcomp50370_c0_seq1) having the most significant change in purple potato (LogFC was 3.54 and 3.50 for skin and 9.4 and 7.65 for flesh respectively). Only one CHI named *CHI1* (PGSC0003DMT400030430) located on chromosome 5 was found to be highly expressed in both skin and flesh of purple potato compared to white potato (1.1 and 4.4 fold change in skin and flesh respectively), and one new transcript *CHI2* (OkPFcomp47529_c0_seq1) was only highly expressed in purple skin (1.23). Six genes annotated as *F3’H* were differentially expressed in skin and flesh of the purple cultivar with two F3’Hs—*F3’H2* (PGSC0003DMT400014232) located on chromosome 4 and *F3’H3* (OkPScomp54523_c2_seq4) were highly expressed in both purple skin (4.13 and 4.96) and purple flesh (5.36 and 6.03), but one *F3'H5* (PGSC0003DMT400016263) located on chromosome 4 was up-regulated in both white skin (-3.02) and white flesh (-3.12). One gene annotated as *F3H* (OkPFcomp51151_c0_seq2) was found in purple skin (2.82) and purple flesh (6.92) to be more highly expressed. Two genes annotated as F3’5’H—*StF3'5'H1-1* (OkPFcomp51142_c0_seq3) and *StF3'5'H2-1* (OkWScomp41567_c0_seq1) were highly expressed both in purple skin (1.16 and 2.01) and purple flesh (1.35 and 7.12). Three genes annotated as *DFR* were found all highly up-regulated in purple cultivar, two *DFR* genes named *DFR1* (PGSC0003DMT400009287) located on chromosome 2 and *DFR3* (OkPFcomp18287_c0_seq1) were highly expressed in both purple skin (2.26 and 2.94) and purple flesh (6.05 and 4) respectively, while *DFR2* (PGSC0003DMT400087830) located on chromosome 10 was only expressed in the purple skin (4.82). One gene annotated as *LDOX/ANS* (OkPScomp56725_c0_seq1) located on chromosome 8 was significantly up-regulated in both PS (2.57) and PF (5.63). 12 genes annotated as *UFGT* were differentially expressed in purple skin and flesh. Of these, seven *UFGTs* were more highly expressed in purple skin and purple flesh, while five *UFGTs* were more highly expressed in white skin and white flesh. The *UFGT1* (OkPFcomp47734_c0_seq1) has been previously studied by Wei [43], who found that over-expressing *UFGT1* can increase anthocyanin content in transgenic potato lines. However, *UFGT6* showed the highest expression in our dataset. One *GST* (PGSC0003DMT400043105) is highly expressed in purple skin (3.37) and purple flesh (8.85).

Of particular interest for understanding the regulation of anthocyanin biosynthesis were the DEGs categorized as TFs. There were 225 TFs up-regulated in anthocyanin rich tubers, of which 49 TFs were up-regulated only in purple skin and 65 TFs up-regulated only in purple flesh, while 111 TFs were up-regulated in both purple skin and purple flesh, 221 were down-regulated in tissues with high anthocyanin, of which 65 TFs were down-regulated only in purple skin and 54 TFs were down-regulated only in purple flesh, while 102 TFs down-regulated in both purple skin and purple flesh (Fig 3b). The DEGs which were identified as transcription factors included members of classes that are implicated in regulating anthocyanin biosynthesis, such as the R2R3 MYB, bHLH and WD40 classes (Table 6). The expression pattern and the DNA-binding specificity of MYB proteins and, to some extent, bHLH proteins also determine the subset of pathway genes that are activated, whereas WD40 proteins appear to have a more general role in the regulatory complex [13].

**Table 6:** The number of up-regulated and down-regulated transcription factors in both PS vs.WS and PF vs. WF libraries.

C2H2 (10,6)*	Dof (7,4)	C3H (8,17)	MADS-box (8,2)	E2F (4,3)	AP2/ERF (9,8)	WD40 (8,8)
FAR1 (1,0)	GATA (2,3)	GRAS (2,4)	HB-other (5,5)	HD-ZIP (2,3)	ZF-HD (0,3)	TCP (0,3)
HSF (1,1)	LSD (0,1)	MYB (9,11)	NAC (8,4)	WRKY (9,0)	bHLH (7,9)	bZIP (11,8)

*The first number in parentheses indicates the number of a particular class of TF up-regulated in PS compared to WS, and also up-regulated in PF compared to WF. The second number indicates the down-regulation in PS compared to WS, and also down-regulation in PF compared to WF.

**Fig 5:**
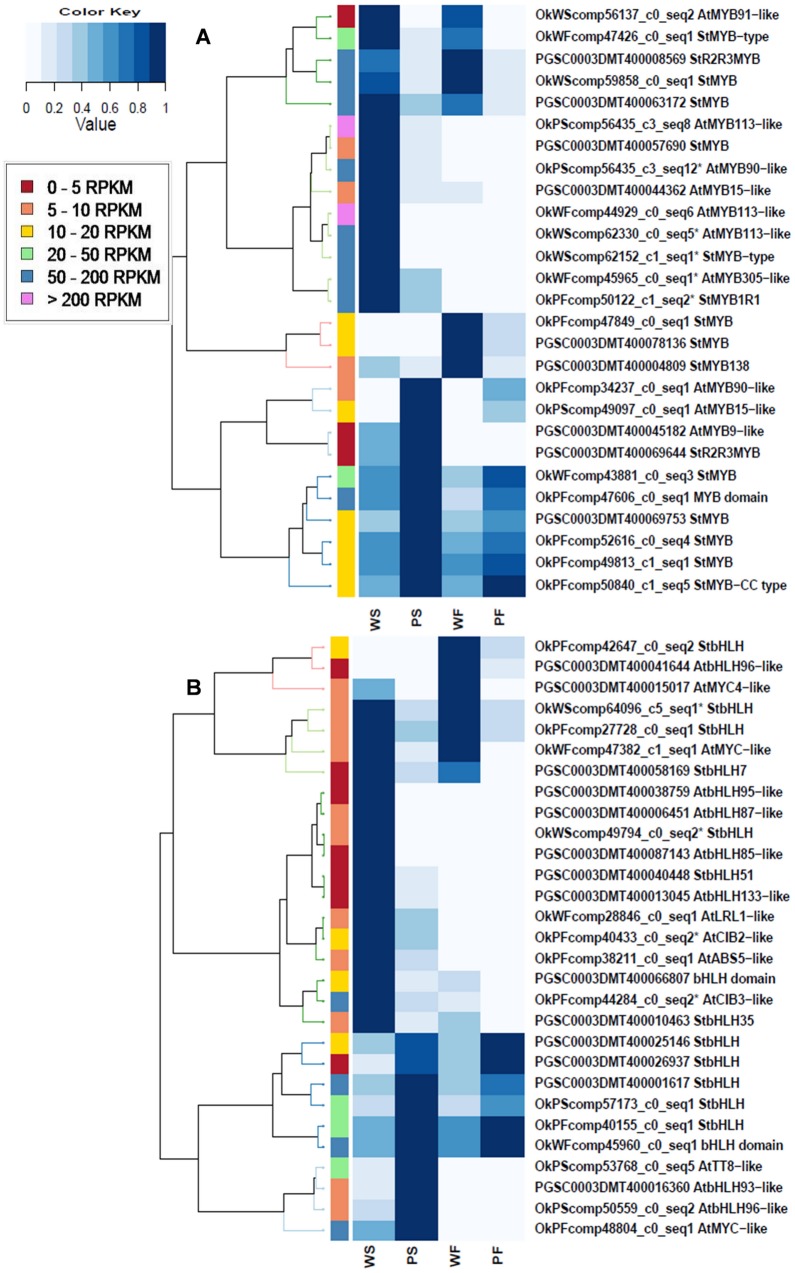
Hierarchical clustering of differentially expressed transcription factors of (A) MYB (B) bHLH in WS, PS, WF, PF libraries. Normalized RPKM values are represented as colors ranging from white (0: little or no expression) to violet (1: highest expression within four libraries). Each column represents a library, each row represents a gene. * indicates partial sequences. The colour bar on the left indicates the range of the highest RPKM value within four libraries for each gene.

There were 26 MYB and 27 bHLH TFs annotated in these classes as being differentially expressed in PS vs. WS libraries, 21 MYB and 17 bHLH TFs were differentially expressed in PF vs. WF libraries (S4 Table and Fig 5). In Arabidopsis, most of the late pathway genes (*DFR*, *ANS*, *UFGT*) are regulated by a MBW complex [44]. One potato MYB (OkPFcomp34237_c0_seq1), which is the best blast match to Arabidopsis *AtMYB90*, was found to be highly up-regulated in purple skin (3.69) and purple flesh (3.47). This gene shares 88.6% identity at the nucleotide level to *StAN1* which regulates potato periderm coloration in potato tubers [45]. Interestingly, the other partial *StAN1*-like MYB gene (OkPScomp56435_c3_seq12*) was highly up-regulated in white skin (-1.99), but not in white flesh. Three MYBs (OkWFcomp44929_c0_seq6, OkWScomp62330_c0_seq5 and OkPScomp56435_c3_seq8) which are the homologous genes of *AtMYB113* in Arabidopsis, were also found all highly up-regulated in white skin (-7.30, -6.44 and -2.42) and white flesh (-7.84, -8.37 and -2.79. These are all homologs of *StMYBA1* which corresponds to the translated sequence of *StAN3*, indicated as a possible *AN1* pseudogene [45]. The results show that *StAN1-1* (OkPFcomp34237_c0_seq1) is the key transcription factor involved in anthocyanin regulation in tuber skin of purple cultivar and it also regulates the flesh coloration since it is also highly expressed in the purple flesh. The function of the *StAN1-2*, *StMYBA1-1*, *StMYBA1-2 and StMYBA1-3* genes is worth further investigation. One bHLH, annotated as *StbHLH1* (PGSC0003DMT400033569) located on chromosome 9, which is homologous to the gene *AtTT8* was highly expressed in both purple skin (2.84) and purple flesh (3.12). There was no differential expression between the two libraries of *StAN11*, which is the homologous gene of the WD40 *AtTTG1* (Table 5).

Apart from these anthocyanin-related TFs, we also found some differentially expressed TFs between white and pigmented libraries which could be potentially related to anthocyanin metabolism such as MADS-Box, WRKY and NAC [46–48] (Table 6 and S4 Table). OkPFcomp50769_c0_seq3 is homologous to the MADS box gene Short Vegetative Phase (SVP; At2g22540) and is up-regulated in pigmented tissues. Over-expression of a kiwifruit a SVP gene suppresses anthocyanin biosynthesis [49]. OkPScomp53774_c1_seq6 is homologous to WRKY44 or TRANSPARENT TESTA GLABRA 2 (At2g37260) which, in Arabidopsis is regulated by phenylpropanoid-related MYBs [50], and it is up-regulated in purple skin and flesh. Recently NAC transcription factors have been implicated in regulation of peach fruit anthocyanins, including homologues of AtNAC100 (At5g61430, [51]). Two potato homologues of AtNAC100 are up-regulated in purple tubers (PGSC0003DMT400050262 and OkPFcomp51484_c0_seq2). Functional studies of these genes will be necessary to further characterize possible regulatory factors of the anthocyanin pathway and pigmentation metabolism.

### Confirmation of Differential Gene Expression by qPCR

**Fig 6:**
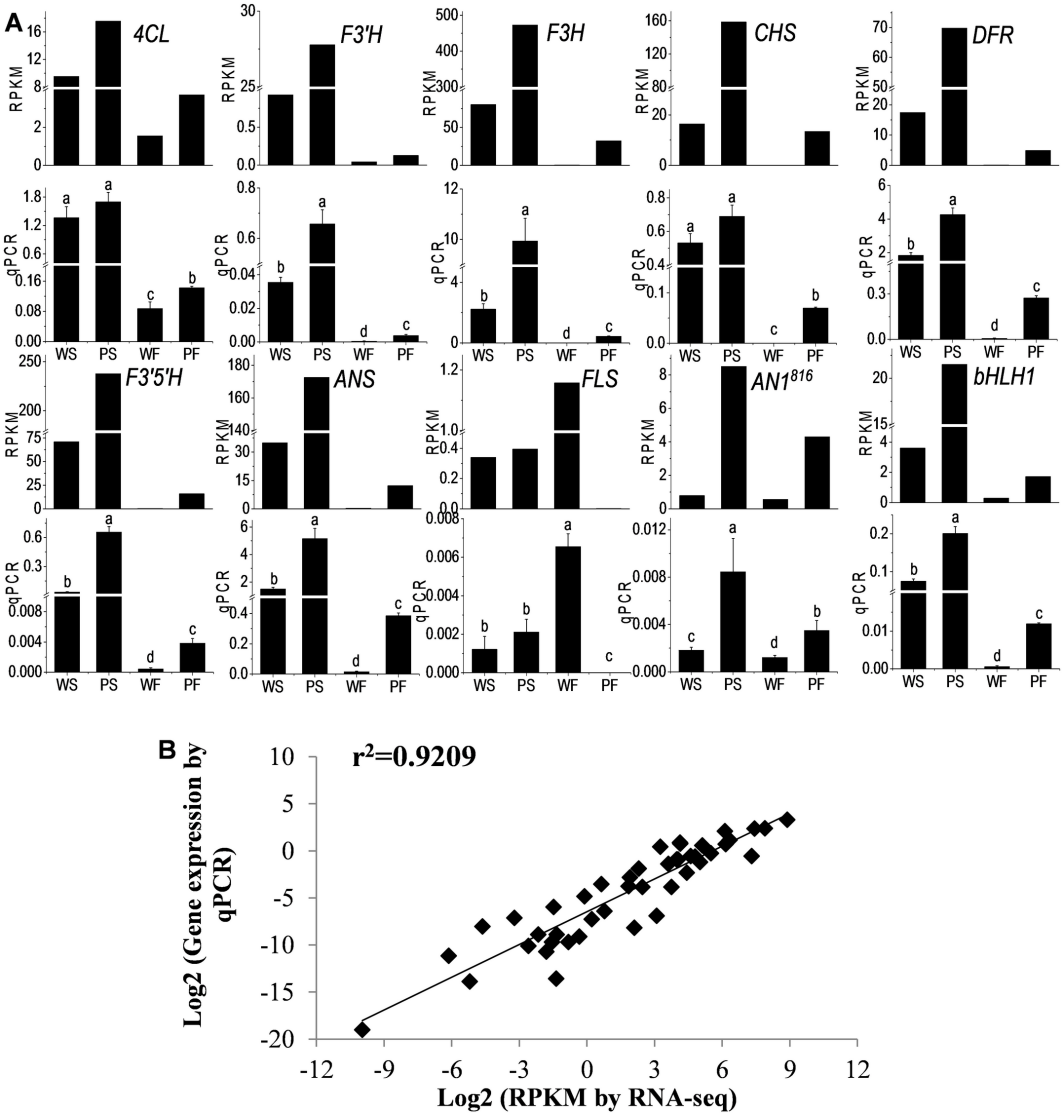
Transcriptomic and qPCR analysis of the genes in anthocyanin biosynthetic pathway and putative transcriptional regulators. (A) transcriptomic and qPCR analysis. RPKM: RPKM obtained by mapping RNA-seq reads to reference genes and *de novo* assembly. qPCR: real time PCR. qPCR data are presented as means (±SE) of four technical replicates. Statistical significance was determined by one-way ANOVA; significant differences between means (LSD, P < 0.05) are indicated where letters (a, b, c, etc.) above the bar differ. *4CL* RPKM: RPKM to PGSC0003DMT400008182; *F3’H* RPKM: RPKM to PGSC0003DMT400063351; *F3H* RPKM: RPKM to OkPFcomp51151_c0_seq2; *CHS* RPKM: RPKM to PGSC0003DMT400049165; *DFR* RPKM: RPKM to PGSC0003DMT400009287; *F3’5’H* RPKM: RPKM to PGSC0003DMT400001124; *ANS* RPKM: RPKM to PGSC0003DMT400058554; *FLS* RPKM: RPKM to PGSC0003DMT400036565; *AN1* ^*816*^ RPKM: RPKM to OkPFcomp34237_c0_seq1; *bHLH1* RPKM: RPKM to PGSC0003DMT400033569. (B) Correlation of gene expression by qPCR and corresponding RNA-Seq analysis.

**Fig 7:**
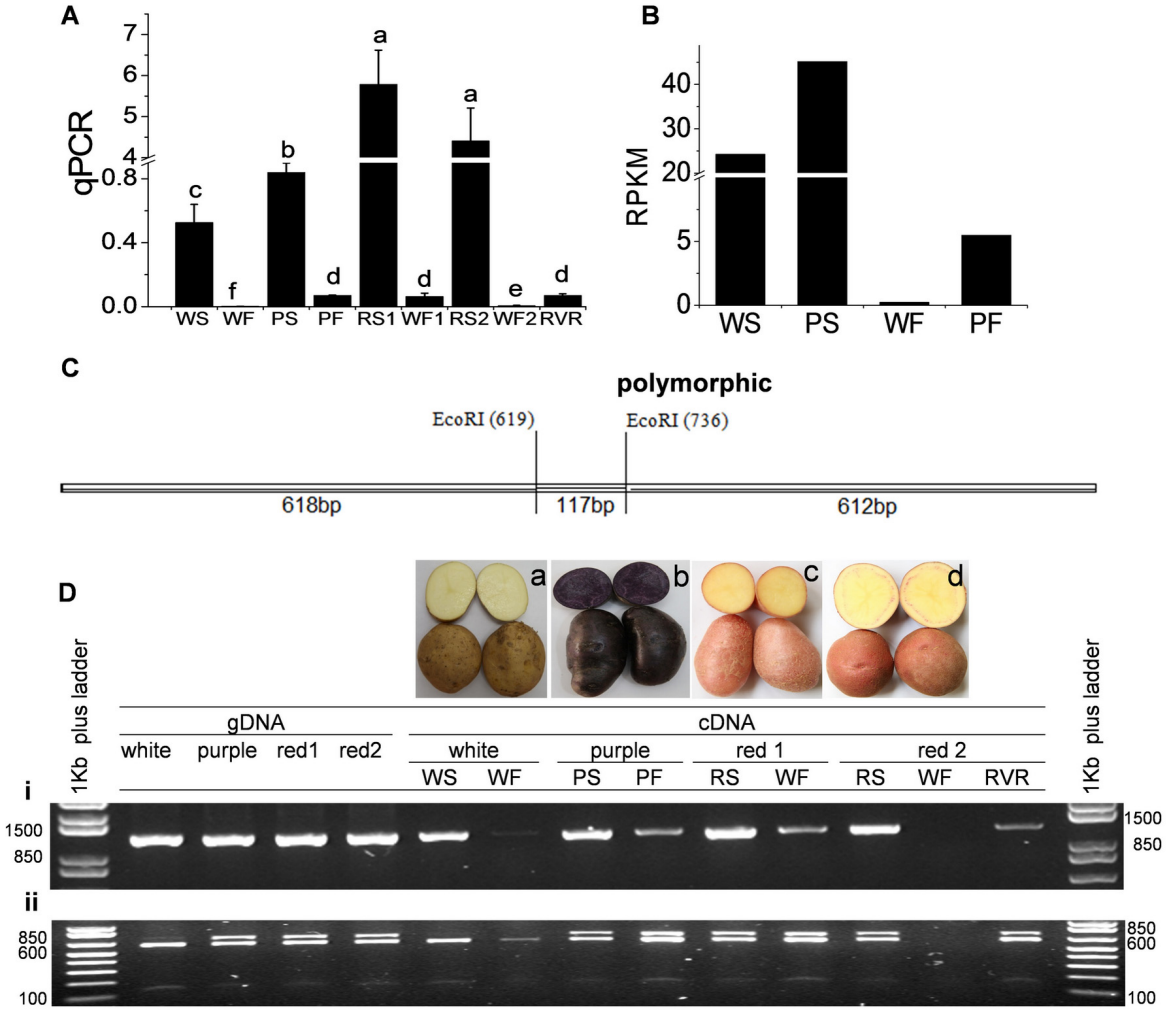
Expression and allelic composition of *UFGT1* in skin and flesh of four potato cultivars. (A) qPCR analysis of *UFGT1* gene expression in skin and flesh of four potato cultivars. Statistical significance was determined by one-way ANOVA; significant differences between means (LSD, P < 0.05) are indicated where letters (a, b, c, etc.) above the bar differ. (B) RNA-seq analysis of *UFGT1*. RPKM: RPKM obtained by *de novo* assembly. RVR: red vascular ring. (C) The diagram of *UFGT1* gene. The position of a polymorphic *Eco*RI restriction site, which differentiates two alleles of *UFGT1* gene in four potato cultivars, is also shown. (D) SNPs validation and allelic composition of *UFGT1*. (i) The full length of genomic DNA and cDNA of *UFGT1* was cloned from four cultivars by PCR, respectively. (ii) PCR products obtained from (i) were digested by *Eco*RI restriction site. (a) white cultivar ‘Xin Daping’, (b) purple cultivar ‘Hei Meiren’, (c) red cultivar ‘Gannongshu NO.5’, (d) red cultivar ‘Qinshu NO.9’.

We further validated 11 differentially expressed genes that, due to previous publications or best blast match to Arabidopsis, are likely to be involved in anthocyanin biosynthesis or the transcriptional regulation of the pathway, using qPCR with gene-specific primers (S1 Table) to confirm their gene expression pattern. The results showed that there was a good correlation between RNA-seq data and qPCR data (Figs 6, 7A and 7B).

Real time qPCR analyses with RNA isolated from the skin and flesh of 7 cultivars revealed that *UFGT1* was highly expressed in the colored skin of all cultivars (Fig 7A and S4 Fig), the RPKM of *UFGT1* was obtained by *de novo* assembly showed that the *UFGT1* expression was 2 times higher in purple skin than that in white skin (Fig 7B). The *UFGT1* expression in flesh was much lower than that in the skin of the 7 cultivars, although it is still well expressed in pigmented flesh (purple flesh of ‘Hei Meiren’ and red vascular ring of ‘Qinshu NO.9’) with almost no expression in white flesh of ‘Xin Daping’, ‘Agria’, ‘Heather’, ‘Gannongshu NO.5’, ‘Qinshu NO.9’ and ‘Red Jackets’.

### SNPs and indel discovery in *MYB AN1*, *bHLH1* and *UFGT* genes

23 SNPs in purple cultivar ‘Hei Meiren’ and 35 SNPs in white cultivar ‘Xin Daping’ were detected with respect to the published *AN1* (AY841127) from red skin of ‘Y83-1’ cultivar (S5 Table). 7 out of 23 (30.4%) of purple cultivar and 13 out of 35 (37.1%) of white cultivar are synonymous SNPs, 14 out of 23 (60.9%) of purple cultivar and 24 out of 35 (68.6%) of white cultivar are transitions. A triple consecutive substitution (TGG to CCA) was found at nucleotide positions 217–219 in both cultivars (100% of total 58 counts in purple cultivar and 99% of total 192 counts in white cultivar) resulting in a substitution from non-polar tryptophan to non-polar proline.

35 SNPs in purple cultivar ‘Hei Meiren’ and 64 SNPs in white cultivar ‘Xin Daping’ were detected in the published *bHLH1* (JX848660) from purple tuber of ‘Magic Molly’ as the reference sequence (S6 Table). 18 out of 35 (51.4%) of purple cultivar and 26 out of 64 (40.6%) of white cultivar are synonymous SNPs, 20 out of 35 (57.1%) of purple cultivar and 42 out of 64 (65.6%) of white cultivar are transitions. A double consecutive substitution (AT to GC) and a triple consecutive substitution (CAT to AGA) were found at nucleotide positions 1475–1476 and 1483–1485 in white cultivar resulted in substitutions from polar asparagines to polar serine and positively charged R group histidine to positively charged R group arginine. We also found a deletion at positions 743–748 (ATGAGG) (7 out of 54 counts) in purple cultivar and a deletion at positions 1731–1733 (GAA) (9 out of 11 counts) in white cultivar, respectively.

There were 42 SNPs in purple cultivar ‘Hei Meiren’ and 36 SNPs in white cultivar ‘Xin Daping’ with respect to the published *UFGT1* (KP096267- OkPFcomp47734_c0_seq1) from the white cultivar Xin Daping’ (S7 Table). There were no indels detected in *UFGT1* gene. We also found a single nucleotide mutation in *UFGT1* from the purple cultivar ‘Hei Meiren’, from T to A in 36 counts out of 52 counts (69%), which results in the loss of an *Eco*RI restriction site at nucleotide position 739. No such mutation was detected in any alleles of the white cultivar ‘Xin Daping’.

Two pairs of primers were designed for cloning the full length genomic DNA and cDNA of *UFGT1* from seven potato cultivars (Fig 1 and S1 Fig), and then the PCR products were digested with *Eco*RI restriction enzyme to validate SNPs and investigate the allelic composition of *UFGT1*. Digestion of PCR products with *Eco*R1 confirmed that all alleles of *UFGT1* in white cultivar ‘Xin Daping’ contained the *Eco*RI restriction site at nucleotide position 735–740 which was consistent with the RNA-seq result (Fig 7 and S4 Fig). However, in other cultivars at least two alleles with or without this *Eco*RI restriction site were present including the other white cultivar ‘Agria’. The function of alleles with or without this mutation requires further investigation.

## Discussion

In this study, a mapping approach was used to align the sequencing data to the potato genome sequence (PGSC), and *de novo* assembly used to assemble new transcripts. The disadvantage of mapping sequence reads to a reference sequence is that structural variation such as chromosome rearrangements, inversions and large (transposon) insertions are likely to be missed. Structural variation between reads and reference genome complicates the mapping of reads [52]. Furthermore, one of the main focuses of this study was to identify genes involved in anthocyanin biosynthesis in white potato ‘Xin Daping’ and purple potato ‘Hei Meiren’. Since the potato reference genome (PGSC) is from DM *Solanum tuberosum* Group Phureja clone DM1-3 516R44 with white potato tubers, this means that some purple cultivar-specific genes may be excluded in the reference gene models. In this study, *de novo* assembly was used to avoid this bias. Grabherr [53] reported that the use of Trinity as a new software for *de novo* transcriptome assembly, and the number of Trinity-assembled transcripts was substantially higher than achieved by other *de novo* assemblers, such as TransABySS, ABySS, and SOAPdenovo. Trinity could reconstruct more transcripts and their variants with high assembly accuracy. Furthermore, Trinity can obtain polymorphic transcripts identified by a small variation (a number of SNPs or small Indels) [54], making it useful to detect the differences in detail between alleles.

Potatoes with red or purple flesh or colored peel are a good source of anthocyanins and as well as being associated with other polyphenols. The peel in particular differs from many other agricultural products because of the presence of nutritionally and pharmaceutically interesting compounds [55]. In the present study, a comparative expression profiling strategy between purple cultivar ‘Hei Meiren’ and white potato cultivar ‘Xin Daping’ was used to identify genes that were differently expressed. Most of the genes implicated in anthocyanin biosynthesis were significantly higher in ‘Hei Meiren’ than ‘Xin Daping’. These included *C4H*, *4CL*, *CHS*, *CHI*, *F3H*, *F3’H*, *F3’5H*, *DFR*, *ANS*, *UFGT* and *GST* (Table 5). The transcript for FLS, an enzyme which generates flavonols, was absent in purple flesh, but higher in white flesh of ‘Xin Daping’. However both RPKM values and qPCR suggested that expression is very low.

The UFGT gene encodes an enzyme that is the final step in catalyzing anthocyanin to anthocyanin-3-glucoside. The transfer of the glucosyl moiety from UDP-glucose to the 3-hydroxyl group of anthocyanidins by UFGT was shown to be the key for anthocyanidin stability and water solubility, it was regarded as an indispensable enzyme of the main biosynthetic pathway to anthocyanins [56]. A candidate potato *UFGT* has been transformed in Arabidopsis, resulting in the accumulation of anthocyanin [57]. Boss et al. [58, 59] showed that the expression of *UFGT* gene was critical for anthocyanin biosynthesis in the grape berry. Hu et al [60] suggested that potato *UFGT* might play a regulatory role in anthocyanin biosynthesis. Wei et al [43] over-expressed *UFGT* (best match to *UFGT1* in Table 5) gene in wild-type potato tuber and found that the tuber color and the anthocyanin content were enhanced noticeably in the transgenic plants. In this study, we confirmed that *UFGT1* (OkPFcomp47734_c0_seq1) correlates with anthocyanin biosynthesis in the potato tubers. In addition, we found another 11 genes annotated as *UFGT* which were differentially expressed in purple skin and flesh (Table 5). However, all the RPKMs of these genes were low in flesh except *UFGT6* (OkPFcomp49428_c0_seq1; RPKM of 180 in purple flesh). Using RNA-seq we discovered SNPs within the *UFGT* gene in purple and white cultivars that might be involved in the functional change, or useful for mapping. For example, the SNP resulting in an *Eco*RI restriction site of *UFGT* at nucleotide position 739 is present in all alleles of white cultivar ‘Xin Daping’ but not the other six cultivars.

Anthocyanin biosynthesis is regulated at the transcriptional level via a set of transcription factors including R2R3-MYB, bHLH, and WD40 [44]. In potato the molecular mechanisms and genes that control anthocyanin accumulation or biosynthesis in the tubers have received much attention [19, 31, 45, 61]. All this work has emphasized the role of the MYB *AN1* in controlling the expression of structural genes involved in the anthocyanin pathway, especially in the tuber periderm. Zhang [31] suggested that the allelic configuration of different loci, such as the bHLH allele, may influence the phenotypes, especially in the tuber flesh, when *AN1* is constitutively expressed. However, marker studies demonstrated that this bHLH was present in all 21 analyzed pigmented tuber tetraploids, and was also present in 21 out of 53 white and yellow-fleshed clones, leading to the conclusion that the bHLH is necessary but not solely sufficient for anthocyanin pigment accumulation. Rommens et al. (2008) [62] over-expressed an R2R3 MYB transcription factor, *StMtf1*, driven by a tuber-flesh specific GBSS promoter in potato and observed mottled tubers with pigmented tuber phloem and periderm cells. Thus they suggested that there are additional unknown factors required for potato tuber anthocyanin accumulation. The complex nature of potato tuber anthocyanin accumulation was further investigated by Taylor et al.(2010) [61]. They identified 27 consistently differentially expressed genes in purple sectors and white sectors from the same tubers combined with a similar study using eight other conventional cultivars and advanced selections with different pigmentation. Of these, 14 were associated with anthocyanin biosynthesis, including *ANS*, *F3H*, *DFR* and *GST*. These were also highly expressed in our purple cultivar ‘Hei Meiren’. Interestingly, other candidate activators such as OkPScomp56435_c3_seq12* (annotated as *StAN1-2*, homolog of *AtMYB90*) were highly expressed in white skin of the white cultivar ‘Xin Daping’, while OkWFcomp44929_c0_seq6 (*StMYBA1-1*, homolog of *AtMYB113*), OkWScomp62330_c0_seq5* (*StMYBA1-2*, homolog of *AtMYB113*) and OkPScomp56435_c3_seq8 (*StMYBA1-3*, homolog of *AtMYB113*) were highly expressed in both white skin and white flesh of ‘Xin Daping’. These results suggest additional unknown factors such as repressors prevent anthocyanin biosynthesis. In our RNA-seq data, three candidate repressors PGSC0003DMT400008569, PGSC0003DMT400063172 and OkWScomp59858_c0_seq1 were highly expressed in white skin (-1.27, -1.18 and -1.72) and white flesh (-2.1, -1.29 and -1.78) of ‘Xin Daping’ with RPKM>100 in white skin and RPKM>70 in white flesh.

Many studies have shown that the third helices of both R2 and R3 of R2R3 MYB are involved in the recognition of a specific DNA consensus sequence [63]. In grape, Hichri et al [64] found that a single amino acid change within the R2 domain of the VvMYB5b transcription factor modulates affinity for protein partners and target promoters selectivity. We found high allelic diversities in AN1, especially in the R2, R3 domain and in bHLH1 in white cultivar ‘Xin Daping’ via RNA-seq. We also found a deletion in the third exon of *AN1* in both the purple cultivar and the white cultivar (S5 Table). The function of these alleles is worth further investigation.

Potato is a highly heterozygous crop, with different populations having novel genes and alleles involved in anthocyanin biosynthesis in the tuber. Therefore, understanding the interaction of different alleles regulating skin and flesh pigmentation is complex. Here, we provide the basis to identify genes likely to be involved in anthocyanin biosynthesis including biosynthetic steps and transcription factors. Future work could involve more RNA libraries of distantly related coloured and non-coloured tetraploid cultivars to further identify specific alleles of genes controlling colour-related traits.

## Conclusions

In this study, we used next-generation sequencing technology and bioinformatics tools to analyze the transcriptome of tetraploid white and purple potato cultivars, by both *de novo* assembly of transcripts and alignment to the published diploid potato genome. We generated 60,930 transcripts, of which 27,754 (45%) were novel transcripts that are not clustered with DM potato reference genes, as well as 9393 alternative transcript forms. This provides an excellent platform for future genetic and functional genomic research. We analyzed differentially expressed genes related to anthocyanin biosynthesis between the cultivars and discovered new versions of the pathway genes and potential transcription factors. In addition, putative SNPs were identified and validated in *UFGT*, *AN1* and *bHLH1*. We used the transcriptome results to study the large *UFGT* gene family which plays a role in anthocyanin biosynthesis in the potato tuber. The present transcriptome analysis provides valuable information regarding anthocyanin biosynthesis in potato, including differential responses of gene family members and regulatory transcription factor candidates. Use of this technology within a breeding population will be potentially even more powerful.

## Supporting Information

S1 Fig Tubers of *Solanum tuberosum* cultivars. (A) ‘Gannongshu NO.5’, (B) ‘Qinshu NO.9’, (C) ‘Agria’, (D) ‘Red Jackets’ and (E) ‘Heather’.
(TIF)

S2 Fig Comparison of gene expression levels between two libraries of WS and PS and two libraries of WF and PF. logCPM is log2 counts-per-million, logFC is log2 fold change.
(TIF)

S3 Fig MapMan metabolic pathway overview of DEGs in (A) PS compared to WS and (B) PF compared to WF libraries. Boxes represent FDR < 0.05 and logFC >1 of expression values of differentially expressed genes. The up-regulated and down-regulated genes are shown in blue and red boxes, respectively. CHO stands for Carbohydrate, TCA stands for Tricarboxylic Acid, OPP stands for Oxidative Phosphorylation Pentose.
(TIF)

S4 Fig Expression and allelic composition of *UFGT1* gene in skin and flesh of three potato cultivars. (A) qPCR analysis of *UFGT1* gene in skin and flesh of three potato cultivars. (e) white cultivar ‘Agria’, (f) red cultivar ‘Red Jackets’, (g) purple cultivar ‘Heather’. Statistical significance was determined by one-way ANOVA; significant differences between means (LSD, P < 0.05) are indicated where letters (a, b, c, etc.) above the bar differ. (B) SNPs identification of *UFGT1*. (i)The full length of genomic DNA and cDNA of *UFGT1* was cloned from three cultivars by PCR, respectively. (ii) PCR products obtained from (i) were digested by *Eco*RI restriction site.
(TIF)

S1 Table Sequence information for primers. (XLSX)

S2 Table Differentially expressed genes between WS vs.PS and WF vs. PF transcriptomes. (XLSX)

S3 Table KEGG pathway enrichment analysis between WS vs. PS and WF vs. PF DEGs. (XLSX)

S4 Table Differentially expressed transcription factors in WS vs. PS and WF vs. PF libraries. (XLSX)

S5 Table Summary of 23 SNPs in purple cultivar ‘Hei Meiren’ and 35 SNPs in white cultivar ‘Xin Daping’ with respect to the published *AN1* (AY841127) from the red cultivar ‘Y83-1’. (DOCX)

S6 Table Summary of 35 SNPs in purple cultivar ‘Hei Meiren’ and 64 SNPs in white cultivar ‘Xin Daping’ with respect to the published *bHLH1* (JX848660) from the purple cultivar ‘Magic Molly’. (DOCX)

S7 Table Summary of 42 SNPs in purple cultivar ‘Hei Meiren’ and 36 SNPs in white cultivar ‘Xin Daping’ with respect to the published *UFGT* (KP096267- OkPFcomp47734_c0_seq1) from the white cultivar ‘Xin Daping’. (DOCX)
