# Supplementary figures and images for: Comparative Transcriptome Analysis of White and Purple Potato to Identify Genes Involved in Anthocyanin Biosynthesis
Source: PLoS One. 2015 Jun 8;10(6):e0129148. doi: 10.1371/journal.pone.0129148 (PMC4459980; doi:10.1371/journal.pone.0129148)

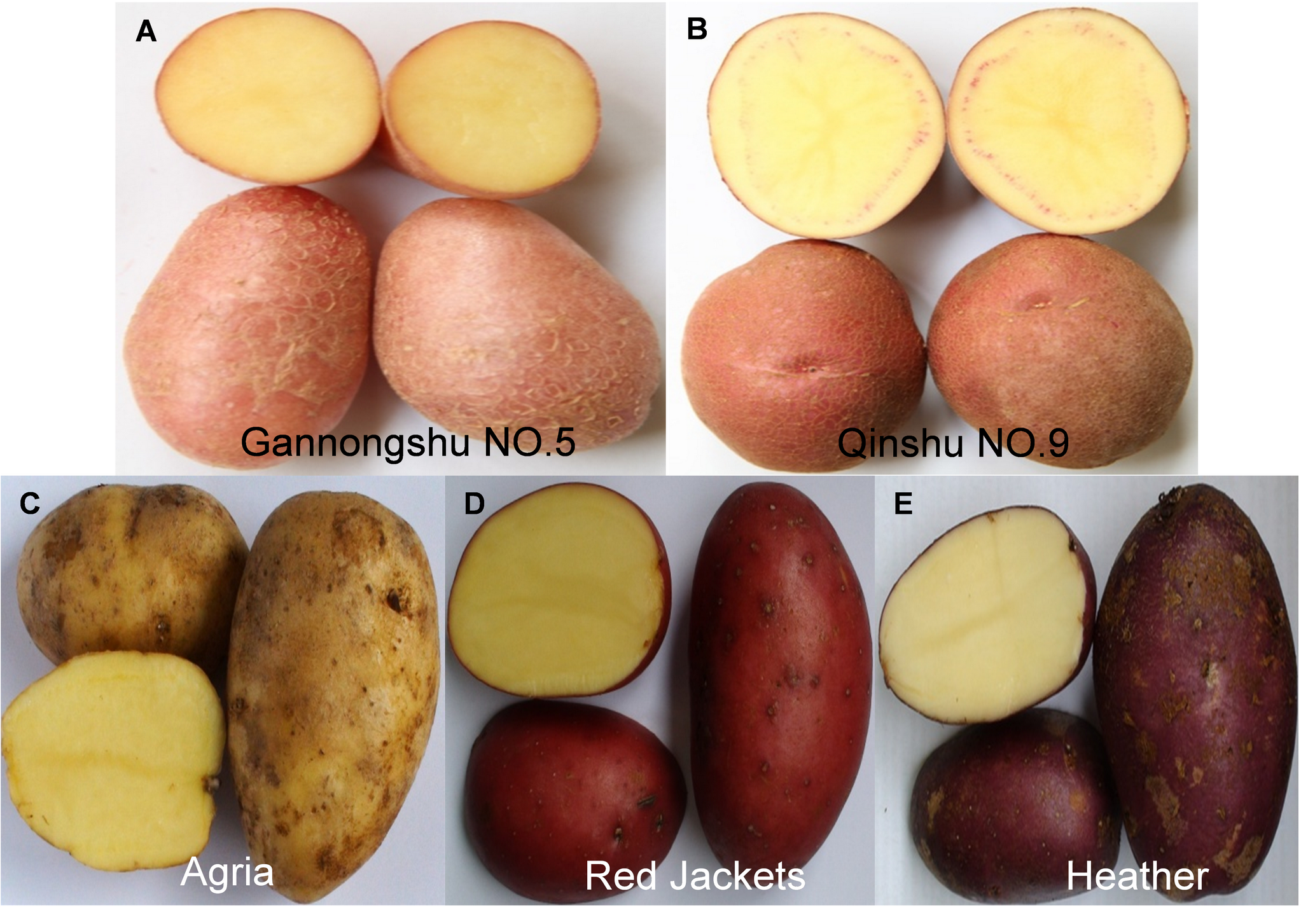

Supplement: S1 Fig — (A) ‘Gannongshu NO.5’, (B) ‘Qinshu NO.9’, (C) ‘Agria’, (D) ‘Red Jackets’ and (E) ‘Heather’. (TIF) [file pone.0129148.s001.tif]

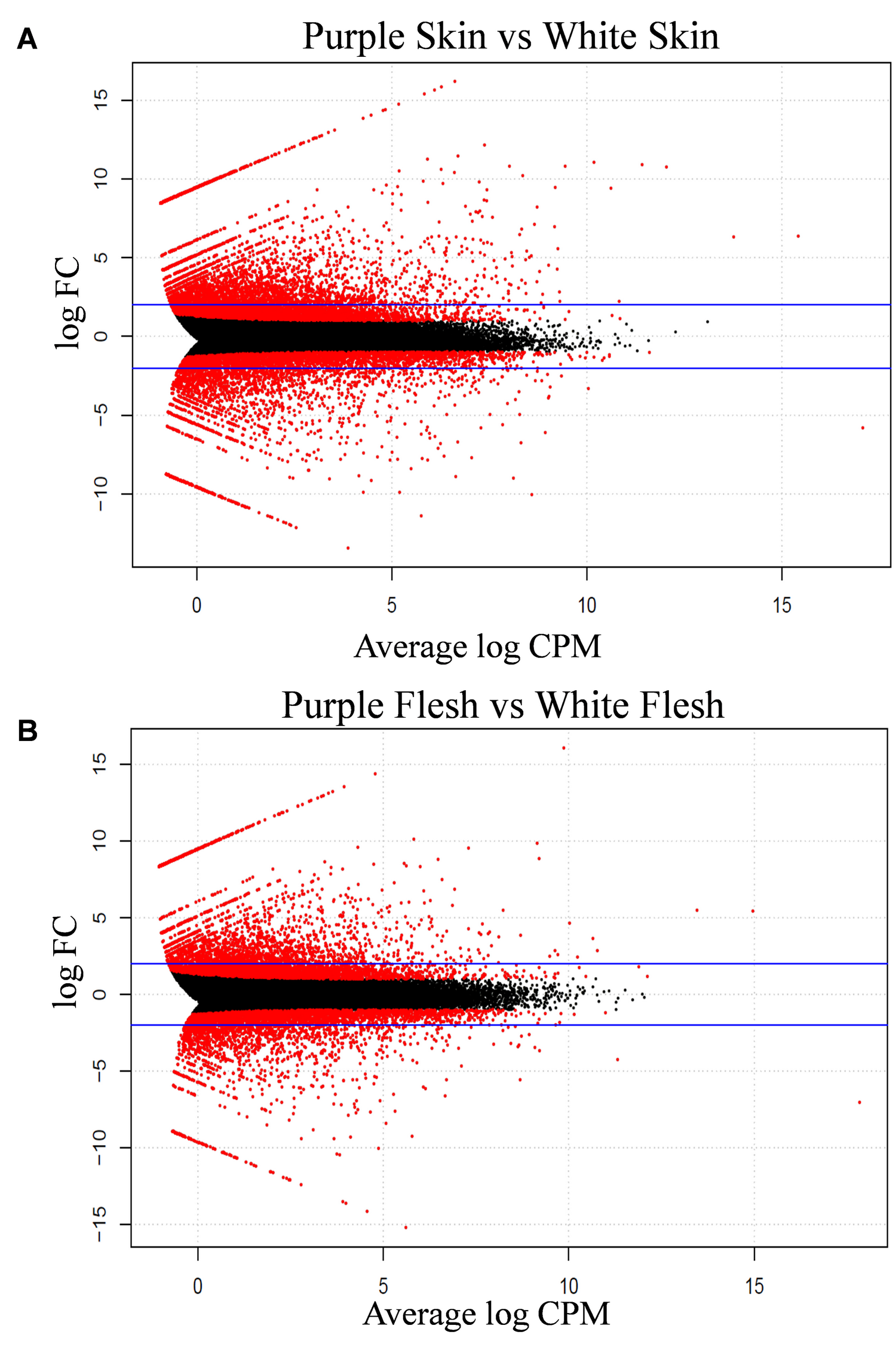

Supplement: S2 Fig — logCPM is log2 counts-per-million, logFC is log2 fold change. (TIF) [file pone.0129148.s002.tif]

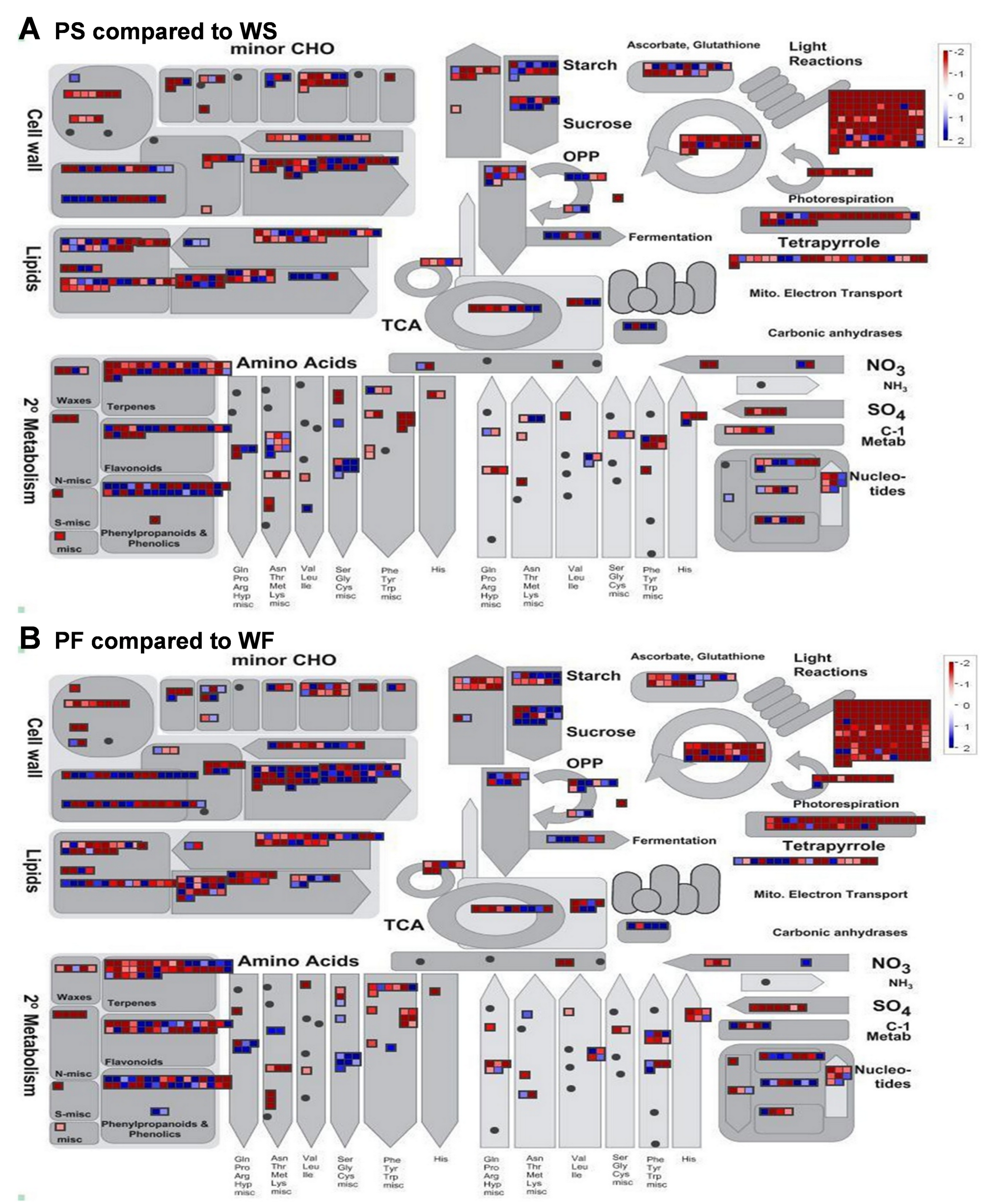

Supplement: S3 Fig — Boxes represent FDR < 0.05 and logFC >1 of expression values of differentially expressed genes. The up-regulated and down-regulated genes are shown in blue and red boxes, respectively. CHO stands for Carbohydrate, TCA stands for Tricarboxylic Acid, OPP stands for Oxidative Phosphorylation Pentose. (TIF) [file pone.0129148.s003.tif]

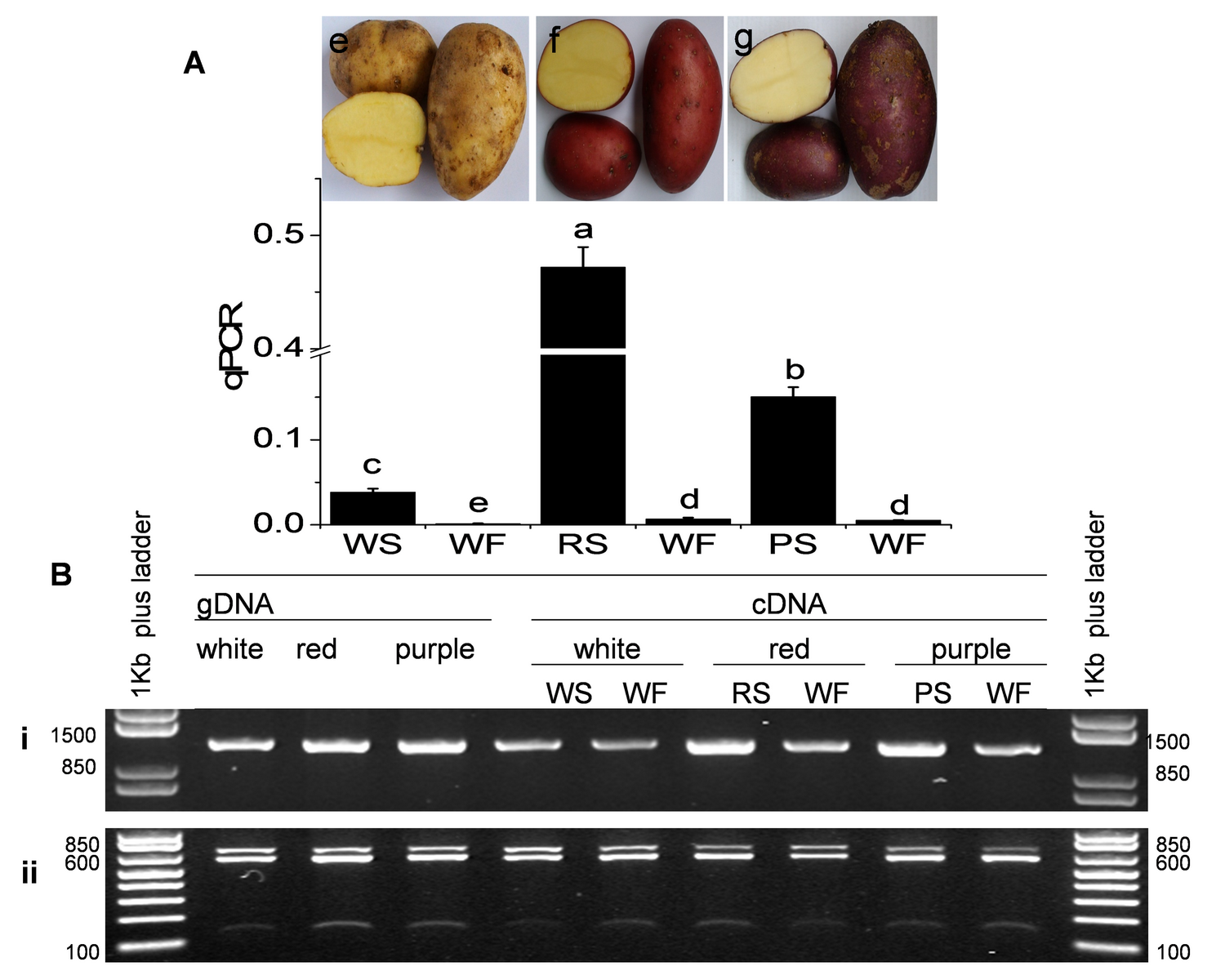

Supplement: S4 Fig — (A) qPCR analysis of UFGT1 gene in skin and flesh of three potato cultivars. (e) white cultivar ‘Agria’, (f) red cultivar ‘Red Jackets’, (g) purple cultivar ‘Heather’. Statistical significance was determined by one-way ANOVA; significant differences between means (LSD, P < 0.05) are indicated where letters (a, b, c, etc.) above the bar differ. (B) SNPs identification of UFGT1. (i)The full length of genomic DNA and cDNA of UFGT1 was cloned from three cultivars by PCR, respectively. (ii) PCR products obtained from (i) were digested by EcoRI restriction site. (TIF) [file pone.0129148.s004.tif]
