# Supplementary material for: Comparative Transcriptome Analysis of White and Purple Potato to Identify Genes Involved in Anthocyanin Biosynthesis
Source: PLoS One. 2015 Jun 8;10(6):e0129148. doi: 10.1371/journal.pone.0129148 (PMC4459980; doi:10.1371/journal.pone.0129148)
Supplement: S5 Table — (DOCX) [file pone.0129148.s009.docx]

**Table S5. Summary of 23 SNPs in purple cultivar ‘Hei Meiren’ and 35 SNPs in white cultivar ‘Xin Daping’ with respect to the published *AN1* (AY841127) from the red cultivar ‘Y83-1’.**

|  | mutations | | Amino acid substitutions | | Total count^*^ | | Mutation rate^**^ (percentage) | |
| --- | --- | --- | --- | --- | --- | --- | --- | --- |
| Nucleotide positions | Purple | white | Purple | white | Purple | white | Purple | white |
| 26 | C>T |  | S>F |  | 28 |  | 100 |  |
| 39 | A>G | A>G | I>M | I>M | 38 | 48 | 8 | 92 |
| 42 | G>A | G>A | R | R | 47 | 91 | 26 | 96 |
| 58 | G>A | G>A | E>K | E>K | 255 | 1212 | 86 | 99 |
| 93 | T>C | T>C | D | D | 289 | 1374 | 87 | 99 |
| 94 |  | A>G |  | K>E |  | 122 |  | 9 |
| 122 |  | T>C | V>A |  |  | 1051 |  | 14 |
| 126 | A>T |  | P |  |  | 899 |  | 99 |
| 127 | A>T | A>T | T>F | T>F | 236 | 899 | 81 | 99 |
| 128 |  | C>T |  | T>S | 235 | 866 | 81 | 99 |
| 133 | A>G | A>G | T>A | T>A | 77 | 166 | 100 | 100 |
| 168 |  | G>A |  | L>L |  | 31 |  | 87 |
| 215 |  | A>C |  | D>A |  | 180 |  | 97 |
| 216 | T>A |  | D>E |  | 58 |  | 98 |  |
| 217 | T>C | T>C | W>P | W>P | 58 | 180 | 100 | 99 |
| 218 | G>C | G>C | W>P | W>P | 58 | 192 | 100 | 99 |
| 219 | G>A | G>A | W>P | W>P | 58 | 193 | 100 | 99 |
| 252 |  | G>A |  | K>K |  | 343 |  | 99 |
| 264 | T>C | T>C | N | N | 41 | 318 | 98 | 5 |
| 265 |  | C>A |  | R |  | 302 |  | 99 |
| 267 |  | A>G |  | R |  | 302 |  | 95 |
| 285 |  | T>G |  | G |  | 305 |  | 5 |
| 300 |  | G>A |  | R |  | 11 |  | 8 |
| 306 |  | T>A |  | A |  | 158 |  | 97 |
| 309 |  | C>T |  | N |  | 156 |  | 66 |
| 318 |  | A>G |  | K |  | 71 |  | 75 |
| 334 |  | A>G |  | N>D |  | 12 |  | 50 |
| 337 |  | C>T |  | L>Q |  | 8 |  | 25 |
| 407-412 | TTGCTC>deletion |  | AP>  deletion |  | 6 |  | 5 |  |
| 405-419 |  | TATTGCTCCTCAACC>  deletion |  | IAPQP> deletion |  | 8 |  | 5 |
| 476 | G>A | G>A | C>Y | C>Y | 116 | 6 | 97 | 33 |
| 526-528 |  | T>G |  | S>A |  | 6 |  | 33 |
| 528 |  | C>G |  | S>A |  | 9 |  | 44 |
| 567 | C>G | C>G | I>M | I>M | 99 | 11 | 98 | 91 |
| 573 | C>T | C>T | N | N | 94 | 9 | 100 | 100 |
| 633 | T>C | T>C | N | N | 35 | 3 | 97 | 67 |
| 695 | G>C | G>C | C>S | C>S | 34 | 5 | 12 | 60 |
| 712 | A>G |  | T>A |  | 24 |  | 79 |  |
| 732 | T>G |  | D>E |  | 22 |  | 82 |  |
| 741 | C>T | C>T | N | N | 22 | 6 | 23 | 83 |
| 747 | G>T | G>T | M>I | M>I | 18 | 6 | 28 | 83 |
| 775 |  | G>A |  | D>N |  | 3 |  | 67 |
| 784 | T>G |  | S>A |  | 8 |  | 88 |  |

* Represents number of reads mapped to the published *AN1* (AY841127) visualized by IGV 2.3.25.

** Represents percentage of mutation rate as a total count.
