# Supplementary material for: Comparative Transcriptome Analysis of White and Purple Potato to Identify Genes Involved in Anthocyanin Biosynthesis
Source: PLoS One. 2015 Jun 8;10(6):e0129148. doi: 10.1371/journal.pone.0129148 (PMC4459980; doi:10.1371/journal.pone.0129148)
Supplement: S6 Table — (DOCX) [file pone.0129148.s010.docx]

**Table S6. Summary of 35 SNPs in purple cultivar ‘Hei Meiren’ and 64 SNPs in white cultivar ‘Xin Daping’ with respect to the published *bHLH1* (JX848660) from the purple cultivar ‘Magic Molly’.**

|  | mutations | | Amino acid substitutions | | Total count^*^ | | Mutation rate^**^ (percentage) | |
| --- | --- | --- | --- | --- | --- | --- | --- | --- |
| Nucleotide positions | Purple | white | Purple | white | Purple | white | Purple | white |
| 48 |  | G>A |  | Q |  | 22 |  | 73 |
| 172 |  | C>T |  | P>S |  | 11 |  | 18 |
| 201 |  | G>T |  | S |  | 2 |  | 14 |
| 246 |  | T>C |  | S |  | 6 |  | 17 |
| 258 |  | A>G |  | S |  | 4 |  | 25 |
| 268 |  | G>A |  | A>T |  | 2 |  | 50 |
| 282 |  | C>G |  | S |  | 4 |  | 25 |
| 309 | G>A | G>A | T | T | 75 | 11 | 63 | 64 |
| 362 |  | T>C |  | I>T |  | 6 |  | 33 |
| 380 |  | C>T |  | A>V |  | 5 |  | 20 |
| 399 |  | C>T |  | H |  | 5 |  | 60 |
| 409 |  | A>C |  | M>I |  | 4 |  | 75 |
| 455 |  | T>G |  | I>R |  | 10 |  | 10 |
| 464 | G>C |  | S>T |  | 50 |  | 6 |  |
| 466 | G>T |  | A>S |  | 52 |  | 8 |  |
| 468 | C>A |  | R>S |  | 53 |  | 8 |  |
| 469 | C>A |  | R>S |  | 52 |  | 6 |  |
| 510 |  | A>G |  | V |  | 15 |  | 33 |
| 587 |  | A>C |  | Q>P |  | 19 |  | 47 |
| 646 | G>A |  | A>T |  | 40 |  | 38 |  |
| 682 |  | C>T |  | P>S |  | 15 |  | 40 |
| 701 |  | G>A |  | C>Y |  | 18 |  | 39 |
| 717 | T>C | T>C | D | D | 100 | 18 | 14 | 50 |
| 718 |  | G>T |  | G>C |  | 18 |  | 39 |
| 743-748 | ATGAGG>deletion |  | ED>  deletion |  | 54 |  | 13 |  |
| 759 |  | G>A |  | E |  | 11 |  | 27 |
| 760 |  | G>A |  | D>N |  | 12 |  | 25 |
| 768 |  | C>T |  | D |  | 21 |  | 24 |
| 817 |  | A>G |  | N>D |  | 22 |  | 41 |
| 880 |  | C>G |  | I>V |  | 6 |  | 83 |
| 887 | C>A | C>A | P>Q | P>Q | 16 | 6 | 19 | 100 |
| 921 | G>C |  | S |  | 14 |  | 21 |  |
| 926 | A>G | A>G | D>G | D>G | 14 | 6 | 29 | 33 |
| 933 | C>G | C>G | G | G | 14 | 7 | 21 | 14 |
| 965 |  | A>C |  | D>A |  | 7 |  | 71 |
| 1065 | T>A | T>A | P | P | 69 | 2 | 52 | 50 |
| 1110 | A>T | A>T | T | T | 82 | 8 | 62 | 63 |
| 1264 | C>G | C>G | H>D | H>D | 197 | 19 | 62 | 42 |
| 1267 |  | G>A |  | G>S |  | 25 |  | 12 |
| 1269 |  | C>T |  | G>S |  | 25 |  | 12 |
| 1276 |  | G>A |  | G>S |  | 24 |  | 17 |
| 1282 |  | G>A |  | G>R |  | 29 |  | 21 |
| 1310 |  | G>A |  | S>N |  | 28 |  | 18 |
| 1360 | C>T | C>T | I | I | 22 | 5 | 36 | 40 |
| 1365 | T>A | T>A | P | P | 21 | 5 | 33 | 40 |
| 1386 | G>A |  | S |  | 13 |  | 15 |  |
| 1393 | T>A | T>A | S>T | S>T | 26 | 7 | 42 | 29 |
| 1404 | C>T | C>T | D | D | 24 | 7 | 50 | 43 |
| 1410 | A>G | A>G | S | S | 26 | 8 | 46 | 63 |
| 1425 | A>G | A>G | R | R | 33 | 9 | 42 | 67 |
| 1436 | G>A | G>A | G>E | G>E | 37 | 10 | 46 | 40 |
| 1443 |  | G>C |  | T |  | 10 |  | 30 |
| 1475 |  | A>G |  | N>S |  | 7 |  | 71 |
| 1476 |  | T>C |  | N>S |  | 7 |  | 71 |
| 1482 |  | C>T |  | N |  | 6 |  | 60 |
| 1483 |  | C>A |  | H>R |  | 5 |  | 20 |
| 1484 |  | A>G |  | H>R |  | 5 |  | 20 |
| 1485 |  | T>A |  | H>R |  | 6 |  | 17 |
| 1533 |  | T>A |  | I |  | 21 |  | 57 |
| 1657 |  | G>A |  | A>T |  | 32 |  | 63 |
| 1688 | A>G | A>G | N>S | N>S | 142 | 32 | 38 | 88 |
| 1696 | A>G | A>G | T>A | T>A | 109 | 32 | 35 | 9 |
| 1700 | T>C | T>C | I>T | I>T | 106 | 32 | 33 | 9 |
| 1710 | A>G | A>G | A | A | 55 | 14 | 25 | 7 |
| 1714 | C>T | C>T | P>S | P>S | 50 | 11 | 22 | 9 |
| 1731-1733 |  | GAA>  deletion |  | K>  deletion |  | 11 |  | 82 |
| 1773 | G>A |  | P |  | 19 |  | 32 |  |
| 1785 | G>C | G>C | T | T | 26 | 7 | 43 | 42 |
| 1787 | T>C | T>C | V>A | V>A | 27 | 7 | 48 | 43 |
| 1789 |  | T>A |  | S>T |  | 7 |  | 43 |
| 1806 | A>G | A>G | E | E | 38 | 6 | 66 | 100 |
| 1849 | T>C | T>C | I | I | 85 | 8 | 78 | 75 |
| 1917 | T>A |  | I |  | 21 |  | 57 |  |
| 1960 | T>C | T>C | F>I | F>I | 54 | 4 | 56 | 100 |
| 1968 | T>G | T>G | F>I | F>I | 67 | 8 | 58 | 100 |

* Represents number of reads mapped to the published *bHLH1* (JX848660) visualized by IGV 2.3.25.

** Represents percentage of mutation rate as a total count.
