# Supplementary material for: Comparative Transcriptome Analysis of White and Purple Potato to Identify Genes Involved in Anthocyanin Biosynthesis
Source: PLoS One. 2015 Jun 8;10(6):e0129148. doi: 10.1371/journal.pone.0129148 (PMC4459980; doi:10.1371/journal.pone.0129148)
Supplement: S7 Table — (DOCX) [file pone.0129148.s011.docx]

**Table S7. Summary of 42 SNPs in purple cultivar ‘Hei Meiren’ and 36 SNPs in white cultivar ‘Xin Daping’ with respect to the published *UFGT* (KP096267- OkPFcomp47734_c0_seq1) from the white cultivar ‘Xin Daping’.**

|  | mutations | | Amino acid substitutions | | Total count^*^ | | Mutation rate^**^ (percentage) | |
| --- | --- | --- | --- | --- | --- | --- | --- | --- |
| Nucleotide positions | Purple | white | Purple | white | Purple | white | Purple | white |
| 5 | C>A | C>A | L>I | L>I | 30 | 28 | 27 | 39 |
| 6 | T>A | T>A | L>Q | L>Q | 33 | 29 | 73 | 62 |
| 75 | A>T | A>T | L | L | 497 | 247 | 28 | 34 |
| 78 | C>T | C>T | V | V | 525 | 263 | 26 | 32 |
| 87 | A>C | A>C | I | I | 525 | 259 | 82 | 31 |
| 138 |  | T>C |  | N |  | 214 |  | 22 |
| 324 |  | A>G |  | K |  | 20 |  | 25 |
| 351 | T>C | T>C | F | F | 68 | 38 | 22 | 29 |
| 356 | C>G | C>G | S>W | S>W | 66 | 39 | 65 | 28 |
| 417 | C>T |  | G |  | 87 |  | 28 |  |
| 439 | T>A | T>A | L>M | L>M | 157 | 83 | 39 | 22 |
| 488 | A>C | A>C | Q>P | Q>P | 161 | 94 | 66 | 21 |
| 519 | C>T |  | D |  | 101 | 31 |  |  |
| 540 | T>A | T>A | A | A | 85 | 48 | 71 | 35 |
| 568 | T>A | T>A | L>M | L>M | 97 | 57 | 78 | 39 |
| 586 | T>A | T>A | L>I | L>I | 124 | 62 | 35 | 38 |
| 603 | T>G | T>G | A | A | 94 | 53 | 18 | 25 |
| 604-606 | G>A | G>A | D>N | D>N | 95 | 55 | 18 | 24 |
| 606 | T>C | T>C | D | D | 92 | 54 | 18 | 24 |
| 670 | C>A |  | L>I |  | 70 |  | 79 |  |
| 708 | A>G |  | L |  | 91 |  | 60 |  |
| 730 | G>A | G>A | D>N | D>N | 54 | 30 | 15 | 30 |
| **739** | **T>A** |  | **S>T** |  | **52** |  | **69** |  |
| 819 | A>G |  | T |  | 153 |  | 63 |  |
| 828 | T>A |  | P |  | 142 |  | 65 |  |
| 840 | T>A | T>A | I | I | 117 | 33 | 21 | 21 |
| 857 | T>C | T>C | V>A | V>A | 117 | 44 | 83 | 23 |
| 869 | G>A | G>A | R>K | R>K | 135 | 55 | 81 | 31 |
| 891 | T>C | T>C | S | S | 158 | 57 | 20 | 23 |
| 891 | T>A |  | S |  | 158 | 57 | 61 |  |
| 922 | A>G | A>G | K>E | K>E | 168 | 57 | 21 | 28 |
| 927 | G>A |  | G |  | 171 |  | 57 |  |
| 945 | G>C | G>C | K>N | K>N | 174 | 54 | 24 | 87 |
| 966 | G>T | G>T | S | S | 128 | 56 | 22 | 16 |
| 972 | G>C | G>C | A | A | 141 | 63 | 52 | 98 |
| 975 | T>C | T>C | P | P | 135 | 67 | 51 | 98 |
| 1017 | A>G | A>G | V | V | 137 | 68 | 27 | 76 |
| 1020 | A>G | A>G | T | T | 126 | 68 | 51 | 98 |
| 1104 | G>A | G>A | L | L | 269 | 128 | 24 | 70 |
| 1127 |  | T>C |  | V>A |  | 95 |  | 34 |
| 1141 | T>C | T>C | L | L | 191 | 103 | 27 | 98 |
| 1192 | G>A | G>A | G>S | G>S | 216 | 98 | 22 | 98 |
| 1203 | T>C | T>C | F | F | 230 | 115 | 26 | 97 |
| 1253 | G>A | G>A | G>E | G>E | 53 | 31 | 62 | 98 |
| 1310 | C>T | C>T | A>V | A>V | 24 | 9 | 29 | 98 |

* Represents number of reads mapped to the published *UFGT* (KP096267- OkPFcomp47734_c0_seq1) visualized by IGV 2.3.25.

** Represents percentage of mutation rate as a total count.
